# Novel insights into brain lipid metabolism in Alzheimer's disease: Oligodendrocytes and white matter abnormalities

**DOI:** 10.1002/2211-5463.13661

**Published:** 2023-06-26

**Authors:** Noe Kawade, Koji Yamanaka

**Affiliations:** ^1^ Department of Neuroscience and Pathobiology, Research Institute of Environmental Medicine Nagoya University Japan; ^2^ Department of Neuroscience and Pathobiology, Nagoya University Graduate School of Medicine Nagoya University Japan; ^3^ Institute for Glyco‐core Research (iGCORE) Nagoya University Japan; ^4^ Center for One Medicine Innovative Translational Research (COMIT) Nagoya University Japan

**Keywords:** Alzheimer's disease, lipid metabolism in brain, myelin, oligodendrocytes, white matter

## Abstract

Alzheimer's disease (AD) is the most common cause of dementia. A genome‐wide association study has shown that several AD risk genes are involved in lipid metabolism. Additionally, epidemiological studies have indicated that the levels of several lipid species are altered in the AD brain. Therefore, lipid metabolism is likely changed in the AD brain, and these alterations might be associated with an exacerbation of AD pathology. Oligodendrocytes are glial cells that produce the myelin sheath, which is a lipid‐rich insulator. Dysfunctions of the myelin sheath have been linked to white matter abnormalities observed in the AD brain. Here, we review the lipid composition and metabolism in the brain and myelin and the association between lipidic alterations and AD pathology. We also present the abnormalities in oligodendrocyte lineage cells and white matter observed in AD. Additionally, we discuss metabolic disorders, including obesity, as AD risk factors and the effects of obesity and dietary intake of lipids on the brain.

## Graphical abstract

The lipid composition of the brain is altered in Alzheimer's disease (AD), and several studies have shown white matter abnormalities and oligodendrocyte dysfunction in AD brains. Therefore, it is speculated that the alteration of brain lipid metabolism is associated with dysfunction of oligodendrocytes and white matter abnormalities in the AD brain. Further analyses are required to define these associations.

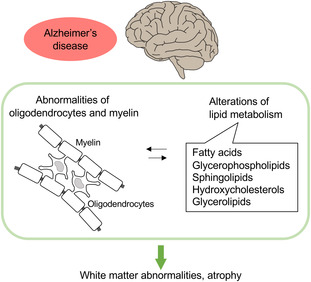

**Abbreviations**
24‐OHC: 24‐S‐hydroxycholesterol
AA: arachidonic acid
ABCA: ATP‐binding cassette subfamily A
AD: Alzheimer's disease
APOE: apolipoprotein E
APOJ: apolipoprotein J
APP: amyloid precursor protein
Aβ: amyloid β
BACE1: beta‐secretase 1
BBB: blood–brain barrier
CE: cholesterol ester
CH25H: cholesterol‐25 hydroxylase
CLDN: claudin
CNPase: cyclic nucleotide phosphohydrolase
CNS: central nervous system
CSF: cerebrospinal fluid
CYP46A1: cytochrome P450 family 46 subfamily A member 1
DAGs: diacylglycerols
DAM: disease‐associated microglia
DAO: disease‐associated oligodendrocyte
DHA: docosahexaenoic acid
EPA: eicosapentaenoic acid
FABPs: fatty acid‐binding proteins
FAS: fatty acid synthase
FAs: fatty acids
FATPs: fatty acid transport proteins
GM1: glycolipid monosialo‐tetrahexosyl‐ganglioside
GWAS: genome‐wide association study
HFD: high‐fat diet
LCPUFAs: long‐chain polyunsaturated fatty acids
LDs: lipid droplets
LXR: liver X receptors
MAG: myelin‐associated glycoprotein
MAGs: monoacylglycerols
MBP: myelin basic protein
MCI: mild cognitive impairment
MOG: myelin oligodendrocyte glycoprotein
MRI: magnetic resonance imaging
MUFAs: monounsaturated fatty acids
NFTs: neurofibrillary tangles
OPCs: oligodendrocyte precursor cells
PCs: phosphatidylcholines
PEs: phosphatidylethanolamines
PICALM: phosphatidylinositol‐binding clathrin assembly protein
PIs: phosphatidylinositols
PLC: phospholipase C
PLTP: phospholipid transfer protein
PPAR: peroxisome proliferator‐activated receptor
PS1: presenilin‐1
PSs: phosphatidylserines
PUFAs: polyunsaturated fatty acids
RXR: retinoid X receptors
SCAP: SREBP cleavage activating protein
SM: sphingomyelin
SREBF: sterol regulatory element‐binding transcription factor
SREBP: sterol regulatory element‐binding protein
TAGs: triacylglycerols
TREM2: triggering receptor expressed on myeloid cells 2
VLCFAs: very long‐chain fatty acids
WMH: white matter hyperintensities

Alzheimer's disease (AD) is the most common cause of dementia. Depositions of amyloid β peptide (Aβ) and hyperphosphorylated tau protein form senile plaques and neurofibrillary tangles (NFTs), respectively, and induce neurodegeneration in the AD brain. Many risk factors for AD have been identified by genome‐wide association studies (GWAS). Several AD risk genes have a role in lipid metabolism. Additionally, epidemiological studies have shown that the levels of some lipid species are altered in the AD brain, suggesting that altered lipid metabolism exacerbates AD pathology. Thus, several studies have recently focused on lipid metabolism in the brain of patients with AD.

Glial cells, such as microglia, astrocytes, and oligodendrocytes, play important roles in the pathological mechanisms underlying neurodegenerative diseases, including AD. Although the number of glial cells is ten times greater than that of neuronal cells, glial cells have been regarded as supporting cells for neurons for a long time. Microglia are among the most studied glial cells, and their neuroinflammatory functions have been widely assessed in various neurodegenerative diseases. On the contrary, the role of oligodendrocytes in those diseases has not been fully clarified. Oligodendrocytes produce the myelin sheath, a lipid‐rich insulator of neuronal axons, and are the main constituents of white matter. Dysfunctions of myelin are related to white matter abnormalities, which have been observed in the brain of patients with AD.

In the present review, we summarize the current knowledge on lipid composition and metabolism in the brain and the myelin. We also review the association between lipid alterations and AD pathology. Additionally, we present the abnormalities in oligodendrocyte lineage cells and white matter found in the AD brain. Because metabolic disorders, including obesity, are known risk factors for AD, we discuss the effects of obesity and dietary intake of lipids on the brain. Lipids and these abbreviations mainly described in this review are summarized in the table (Tables 1, 2, 3, 4).

**Table 1:** Fatty acids (FAs) and these abbreviations described in this review.

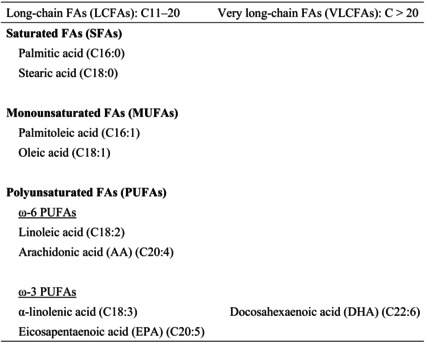

**Table 2:** Glycerophospholipids and these abbreviations described in this review.

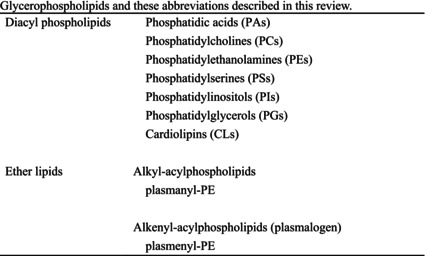

**Table 3:** Sphingolipids and these abbreviations described in this review.

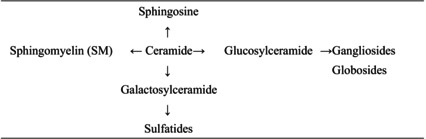

**Table 4:** Sterols and glycerolipids and these abbreviations described in this review.

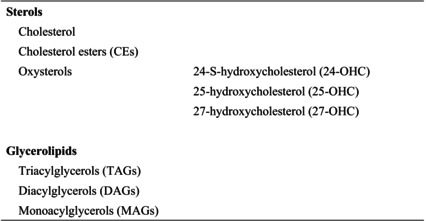

## Lipid composition of the brain

**Fig. 1:**
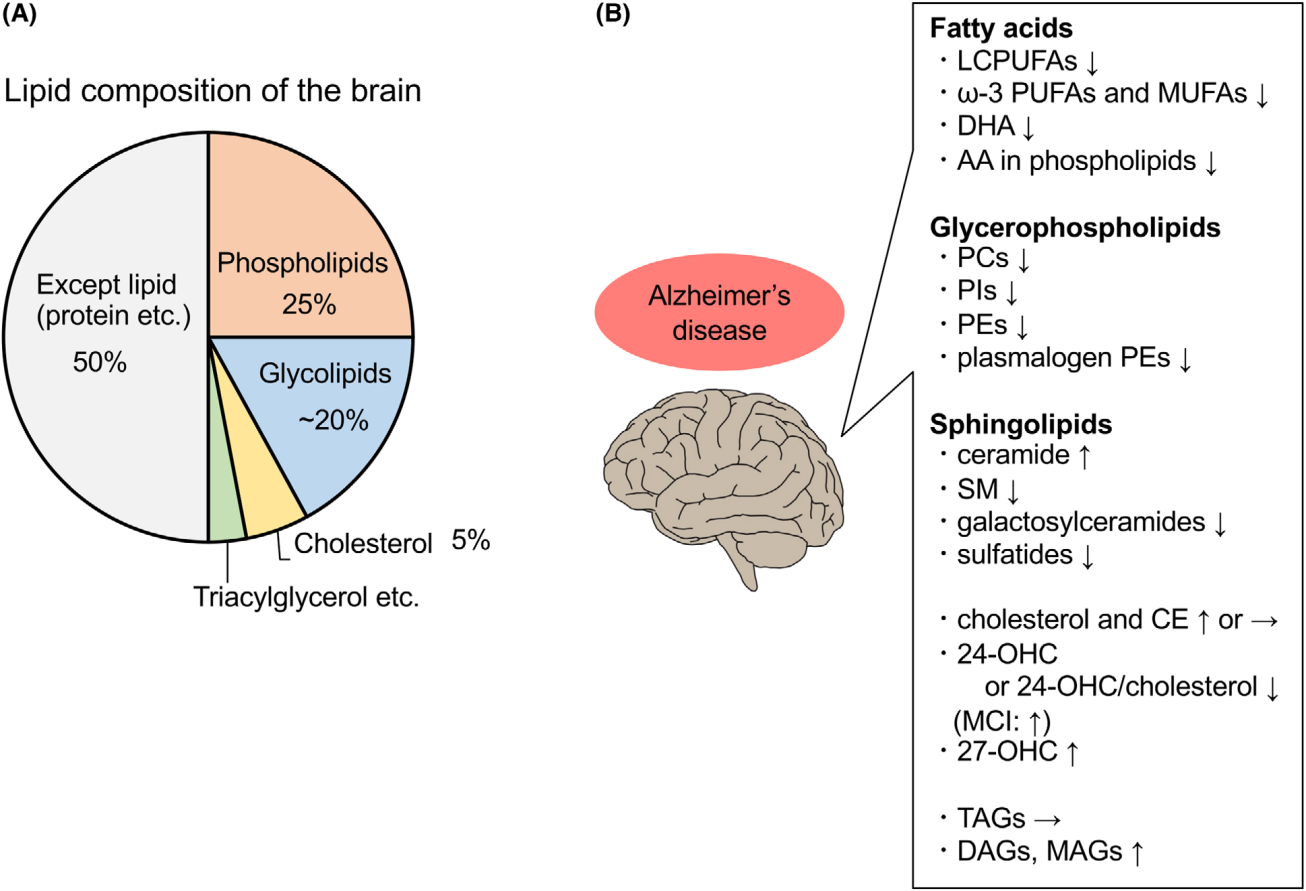
Lipid composition of the brain and alterations in lipid levels in the AD brain. (A) Lipid composition of the brain. The brain is a lipid‐rich organ, and at least 50% of the brain dry weight is lipids. In the brain, lipids comprise 50% phospholipids, below 40% glycolipids, 10% cholesterol and CEs, and traces of triacylglycerol. The figure was created based on reference [2]. (B) Levels of several lipid species are altered in the brain of patients with AD.

The brain is the second most lipid‐rich organ after adipose tissue, and at least 50% of the brain dry weight composition is lipids [1]. Lipids in the brain comprise 50% phospholipids, below 40% glycolipids, 10% cholesterol, cholesterol ester (CE), and traces of triacylglycerol [2] (Fig. 1A).

### Fatty acids

The synthesis of phospholipids and glycolipids needs fatty acids (FAs) (Table 1). The brain produces mostly saturated FAs (SFAs), but it also synthesizes low amounts of polyunsaturated FAs (PUFAs). Thus, most PUFAs in the brain are supplied from peripheral blood by passive diffusion or by mechanisms mediated by adenosine triphosphate‐dependent transporter proteins [1, 3, 4]. However, the transcriptional machinery for PUFA biosynthesis and long‐chain PUFA (LCPUFA)‐containing phospholipid remodeling is present in the cerebral cortex and is stimulated by dietary intake (low supply of precursors) or hormones (high external demands during pregnancy) [5]. LCPUFAs are the source of eicosanoids and docosanoids, which mediate neuroprotective and anti‐inflammatory functions [6]. PUFAs, including docosahexaenoic acid (DHA), modulate synaptic plasticity and neurotransmission [1, 7]. Moreover, FAs have important roles as energy substrates and bioactive molecules. FA oxidation (β‐oxidation) accounts for approximately 20% of the total energy requirements in the brain [8].

### Glycerophospholipids

Glycerophospholipids are the main species of phospholipids (Table 2). As in other tissues, phosphatidylcholines (PCs) and phosphatidylethanolamines (PEs) are the main components of cellular membranes, and anchor membrane proteins in the brain [1, 9, 10]. PCs and PEs participate in determining the stability, permeability, and fluidity of neural membranes, and alterations of PC and PE compositions lead to neurological diseases [11]. Additionally, the degradation of glycerophospholipids produces second messengers such as LCPUFAs [11, 12].

### Sphingolipids

Sphingolipids (sphingophospholipid and sphingoglycolipid) are also components of cellular membranes, and are involved in neurogenesis and synaptogenesis [13, 14] (Table 3). Sphingolipids in synaptic membranes regulate the activity of neurotransmitter receptors [15]. Sphingomyelin (SM), galactosylceramides, and sulfatides are important components of myelin (mentioned in a later chapter). Gangliosides exist in the central nervous system (CNS) at high levels and are associated with cell signaling and neuroprotection [14].

### Cholesterol

The brain also contains high levels of cholesterol compared with those in other tissues, and 25% of the whole‐body cholesterol is in the brain [10, 16] (Table 4). Most sterols in peripheral blood cannot cross the blood–brain barrier (BBB); therefore, they are synthesized in the CNS. The rate of sterol exchange between the brain and peripheral tissues per day is estimated to be lower than 1% [1, 4]. Cholesterol is synthesized predominantly in astrocytes and transferred to neurons through lipoproteins containing apolipoprotein E (APOE). In the brain, cholesterol mainly exists in myelin sheaths, and most of the cholesterol is in an unesterified form, whereas CE accounts for only 1% of the cholesterol stored in lipid droplets (LDs) [17].

Cholesterol is converted into the oxysterol 24‐S‐hydroxycholesterol (24‐OHC), the main free cholesterol species in the brain, by cytochrome P450 family 46 subfamily A member 1 (CYP46A1). CYP46A1 is highly expressed in neurons including pyramidal cells of the cerebral cortex and Purkinje cells of the cerebellum [18, 19, 20]. Around 80% of the whole‐body 24‐OHC is distributed and produced in the brain [21, 22]. 24‐OHC can cross the BBB and is released into the bloodstream, thus preserving cholesterol homeostasis in the CNS [16]. Cholesterol in the brain also converted into 27‐OHC by CYP27A1, and then into 7α‐hydroxy‐3‐oxo‐4‐cholestenoic acid by CYP7B1. CYP27A1 is ubiquitously expressed, but is expressed in neurons, astrocytes, and oligodendrocytes at the low level [19, 20, 21, 23]. 27‐OHC is a major cholesterol metabolite in periphery. Therefore, 24‐OHC is exported from the brain into the periphery, while 27‐OHC enters into the brain [24]. The 27‐OHC:24‐OHC ratio is 1 : 8 in the frontal cortex, 1 : 5 in the occipital cortex, and 1 : 10 in the basal ganglia [25]. 25‐OHC, another oxysterol produced by cholesterol‐25 hydroxylase (CH25H) [26], is enriched in macrophages, dendritic cells, and microglia [27, 28, 29]. The expression of CH25H is induced by inflammatory response [28, 30].

## Alterations of lipid levels in the brain of patients with AD

In the brain of patients with AD, the levels of lipid species are altered. Most of these changes were observed at early disease stages and/or in brain regions initially affected by AD pathology [9] (Fig. 1B).

### Fatty acids

The levels of LCPUFAs, particularly in lipid rafts, were decreased in the brain of patients with AD and mouse models of AD, which leading to abnormalities in nerve cell membranes and pro‐amyloidogenic processing. Such reduction in LCPUFAs is also caused by aging, but is exacerbated in AD pathology [1, 31]. Levels of unsaturated FAs, including those of ω‐3 PUFAs and monounsaturated FAs (MUFAs; primarily oleic acid), are decreased in the brain of patients with AD [32, 33]. The composition in FAs of lipid rafts is characterized by low levels of ω‐3 PUFAs and MUFAs in the cerebral cortex of patients with AD [9, 34] and in the entorhinal and frontal cortices of patients with early‐stage AD [35]. DHA, a ω‐3 PUFA, is the most abundant PUFA in the brain. The levels of DHA are lower in the AD brain, particularly in vulnerable regions such as the hippocampus [36, 37, 38]. Moreover, DHA content in the cerebrospinal fluid (CSF) has been positively correlated with cognitive performance [39]. The level of arachidonic acid (AA), a ω‐6 PUFA, in phospholipids is decreased in the hippocampus of patients with AD [36].

### Glycerophospholipids

Levels of glycerophospholipids including PCs [40, 41], phosphatidylinositols (PIs) [42, 43], and PEs [40, 41], are decreased in the brain of patients with mild cognitive impairment (MCI) and AD, particularly in vulnerable regions such as the hippocampus and cerebral cortex. The levels of plasmalogen PEs are lower in the white and gray matters of the AD brain [44].

### Sphingolipids

Ceramide is a key molecule for the synthesis, recycling, and degradation of other sphingolipids. Ceramide levels are increased in early‐stage AD brains, particularly in the frontal and temporal cortices [45, 46, 47]. SM, galactosylceramides, and sulfatides are important components of myelin (mentioned in a later chapter). SM levels are lower in the AD brain, but this decrease has been observed at a specific disease stage and in a specific brain region [48, 49, 50]. Galactosylceramide levels are also decreased in the hippocampus at early AD stages, before the apparition of tau pathology [51]. Sulfatides are also derived from ceramide. Sulfatide levels are lower in both gray and white matters of the cerebral cortex at the prodromal and early AD stages [47, 52]. These decreases in sphingolipid levels have been involved in myelin degeneration and loss of white matter integrity in the AD brain.

### Cholesterol

Most studies have suggested that cholesterol levels are increased in the brain and blood of patients with AD [25, 45, 53, 54]. Brain cholesterol levels have been positively correlated with the severity of AD [45], and higher cholesterol levels were observed in the cores of senile plaques in the human brain [55]. Moreover, the levels of total CE are increased by more than 1.8‐fold in the entorhinal cortex of patients with AD and in AD mouse models [56, 57]. However, some studies reported no change in cholesterol levels in the human AD brain [58, 59]. It was also reported that the levels of 24‐OHC or 24‐OHC/cholesterol were decreased in the AD brain [20, 25, 58, 60]. In addition, one of these studies showed an increase in 27‐OHC in the AD brain [25]. On the contrary, the level of 24‐OHC was increased in the postmortem brain of patients with MCI [58]. The gene expression of CH25H, an enzyme producing 25‐OHC, was increased in the brains of patients with AD [61] and AD mouse models [62, 63, 64].

### Triacylglycerols, diacylglycerols, and monoacylglycerols

The levels of triacylglycerols (TAGs), diacylglycerols (DAGs), and monoacylglycerols (MAGs) in the brain of patients with AD have also been investigated (Table 4). Although TAG levels are not altered in AD brains, MAG and DAG levels are increased in the frontal cortex of patients with MCI and AD [56, 65, 66].

### Lipidome analysis in Alzheimer's disease mouse models

Recently, lipidome analyses have been conducted using human and mouse brains. Lipidome analyses can comprehensively identify the alterations in the levels of tens of thousands of lipids. Several lipidome analyses revealed that levels of some lipid species are altered in the brain of AD mouse models. However, these alterations are inconsistent among models and/or disease stages. In lipidome studies using amyloid precursor protein (APP)/presenilin‐1 (PS1) [67, 68], Tg2576 x JNPL3 [57], and App^NL‐G‐F^ mice [69], levels of some FAs, eicosanoids, glycerophospholipids, sphingolipids, DAGs, and TAGs were altered in the brain. In the future, and despite their complexity, the results of these lipidome analyses will provide important information to identify new lipid‐linked pathological mechanisms involved in AD.

## Association between lipid metabolism in the brain and Alzheimer's disease pathology

### Alzheimer's disease risk factors mediating lipid metabolism

Alzheimer's disease risk genes have been identified by GWAS, and some of these genes are involved in lipid metabolism. The genetic variant of *APOE* encoded by the *APOE ε4* allele is a well‐known risk factor for AD and is associated with changes in cholesterol and sphingolipid metabolisms [70, 71]. APOE is the main cholesterol carrier and binds to Aβ peptides in the brain to promote Aβ clearance [72]. In *Apoe* knockout mice, cholesterol biosynthesis is reduced, leading to low levels of brain cholesterol [73]. Moreover, aging results in impaired cognitive functions of *Apoe* knockout mice [74]. Triggering receptor expressed on myeloid cells 2 (*TREM2*), apolipoprotein J (*APOJ*) (*CLU*), phosphatidylinositol‐binding clathrin assembly protein (*PICALM*), ATP‐binding cassette subfamily A member 1 (*ABCA1*), and *ABCA7* are risk genes for sporadic AD [9]. APOJ (CLU), PICALM, ABCA1, and ABCA7 mediate lipid transport. TREM2 is expressed on the surface of microglia and can bind to Aβ, lipids, and lipoproteins. TREM2 has been associated with the clearance of myelin debris and remyelination by regulating cholesterol esterification and metabolism of LDs in microglia [75, 76]. Sterol regulatory element‐binding transcription factor 2 (*SREBF2*) is also considered a genetic risk factor for AD [9] and encodes sterol regulatory element‐binding protein 2 (SREBP2), a key regulator of cholesterol metabolism. Overexpression of SREBP2 in APP/PS1 mice stimulates cholesterol synthesis and induces oxidative damage, amyloid accumulation, neuroinflammation, cognitive decline, tau hyperphosphorylation, and NFT formation [77]. Conversely, the genetic ablation of SREBP2 in astrocytes reduces Aβ and tau pathologies [78].

### Fatty acid metabolism in Alzheimer's disease brain

Fatty acid metabolism is also altered in the AD brain. The levels of palmitic acid (C16) are increased and FA synthase (FAS) protein expression is upregulated in the brain of APP/PS1 mice [79]. FAS protein levels are also increased in the cerebral cortex of patients with AD, especially in regions surrounding amyloid plaques [79, 80]. Additionally, acetyl CoA carboxylase, another key enzyme of FA synthesis, is activated in the brain of mouse models of familial AD [81]. Peroxisome proliferator‐activated receptor α (PPARα) is a nuclear receptor positively related to β‐oxidation. The mRNA levels of PPARα are significantly lower in AD brains [82]. Although β‐oxidation occurs primarily in mitochondria, it also takes place in the peroxisomes, specifically for very long‐chain fatty acids (VLCFAs, ≧ C20) [83]. In the brain of patients with advanced AD, VLCFAs accumulate and the volume of peroxisomes in the soma of neurons is increased, and a loss of peroxisomes in neuronal processes occurs with tau hyperphosphorylation [84]. In 3 x Tg mice, LD accumulation has been observed in ependymal cells in the subventricular region, and the proliferation of neural stem cells is suppressed [85]. Several lipid‐sensitive nuclear receptors such as liver X receptors/retinoid X receptors (LXR/RXR), PPARγ, and PPARα have been associated with AD pathology and are potential therapeutic targets [9].

### Lipids and amyloid pathology

Several types of lipids have been involved in the formation of Aβ depositions. Phospholipid transfer protein (PLTP) is associated with lipid and lipoprotein metabolism. PLTP deficiency results in lower levels of PEs and phosphatidylserines (PSs) in the brain, increased intracellular accumulation of Aβ, and memory dysfunction in AD [86]. Phospholipase C (PLC) is an enzyme associated with phospholipid metabolism, especially hydrolysis of phosphatidylinositol‐4,5‐bisphosphate. PLC inhibition decreases the turnover of phosphatidylinositol‐4, 5‐bisphosphate, thus reducing the secretion of Aβ42 [87]. Sphingolipid metabolism has also been associated with the formation of Aβ42 oligomers. Accumulation of SM inhibits γ‐secretase activity and, consequently, a reduction in Aβ secretion [88, 89]. Glycosphingolipids have been linked with the formation of amyloid fibrils [90]. Glycolipid monosialo‐tetrahexosyl‐ganglioside (GM1) binds to released Aβ to form a GM1–Aβ complex in the brain of patients with AD, and the level of GM1–Aβ complexes in the CSF is correlated with the levels of Aβ oligomers [91]. Additionally, cholesterol in lipid rafts contributes to reducing the distance between APP and beta‐secretase 1 (BACE1) before rapid endocytosis [92, 93]. It has also been reported that cholesterol is involved in the activation of BACE1 and γ‐secretase [94]. In addition, brain cholesterol affects tau pathology. The increase in tau phosphorylation and aggregation has been linked with high cholesterol levels in the brain and with a high dietary intake of cholesterol [95, 96]. 24‐OHC is also related to Aβ pathology, but both positive and negative effects were reported. 24‐OHC promotes or suppresses Aβ production, which was presumably dependent on its concentration in the cells [20]. 27‐OHC increases the Aβ deposition in the brain by regulating the production, transportation, and elimination of Aβ [97].

## Lipid composition and metabolism of myelin and oligodendrocytes

**Fig. 2:**
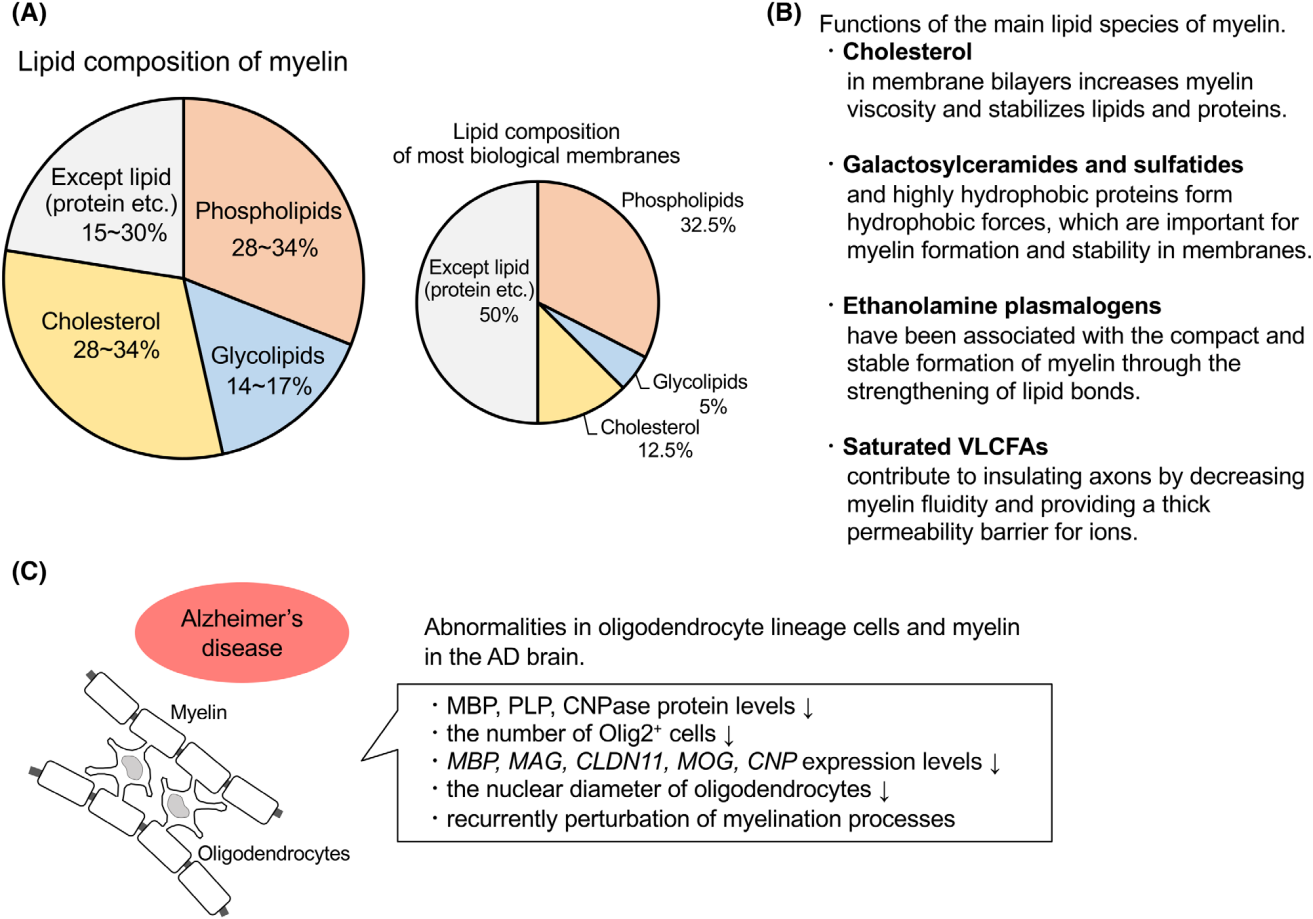
Lipid composition and functions of myelin and alterations in oligodendrocytes and myelin in the AD brain. (A) Lipid composition of myelin. The myelin sheath contains high levels of lipids (70–85%) and low protein levels (15–30%). Lipids in the myeline sheath comprise 40% cholesterol, 40% phospholipids, and 20% glycolipids. In contrast, most biological membranes contain the same levels of proteins and lipids, and the lipid composition is 25% cholesterol, 65% phospholipids, and 10% glycolipids. The figure was created based on reference [98]. (B) Functions of the main lipid species of myelin. (C) Oligodendrocyte lineage cells and myelin are negatively affected in patients with AD.

Oligodendrocytes are kind of glial cells in the CNS and play an important role in producing the myelin sheath. Although most biological membranes contain the same amounts of proteins and lipids, myelin sheaths contain high levels of lipids (70–85%) and low protein levels of proteins (15–30%). The lipid composition in myeline sheaths is 40% cholesterol, 40% phospholipids, and 20% glycolipids (Fig. 2A). In contrast, most biological membranes comprise 25% cholesterol, 65% phospholipids, and 10% glycolipids. Cholesterol, galactosylceramides, and plasmalogens are the major lipid components of myelin and represent 65% of the total lipids in myelin [98, 99, 100, 101] (Fig. 2B).

### Cholesterol

Cholesterol in myelin would be mainly provided by oligodendrocytes and astrocytes [102, 103, 104, 105]. Cholesterol in membrane bilayers increases myelin viscosity and stabilizes lipids and proteins [98, 106]. Moreover, cholesterol is needed for synthesizing myelin during maturation of the CNS, and its availability is a limiting factor for the growth of myelin membranes [105].

### Galactosylceramide

Galactosylceramides and sulfatides, which are sulfated forms of galactosylceramides, represent 20% of total myelin lipids in oligodendrocytes [107]. Galactosylceramides exist preferentially in compact myelin, whereas sulfatides are mainly found in noncompact myelin [108]. In myelin membranes, galactosylceramides and highly hydrophobic proteins form hydrophobic forces, which are important for myelin formation and stability [109, 110]. However, galactosylceramides are not essential for myelin formation, and other glycolipids, such as glucosylceramides, can be produced as partial substitution [109, 111].

### Plasmalogen

Ethanolamine plasmalogens are the main phospholipids in myelin [98]. Although their functions have not been clarified, plasmalogens have been associated with the compact and stable formation of myelin through the strengthening of lipid bonds [44, 109]. Moreover, it has also been suggested that plasmalogens protect myelin against oxidative stress associated with aging [112].

### Fatty acids and myelin

Fatty acids are among the lipids, which, besides cholesterol, are abundantly required to assemble and maintain the formation of myelin. Thus, myelinating cells are vulnerable to the depletion and dysregulation of FAs and lipids [113, 114]. The most abundant FAs in myelin are saturated VLCFAs. Myelin comprises a higher percentage of VLCFAs than that in other plasma membranes [113, 115]. VLCFAs have over 20 carbons and are synthesized in the endoplasmic reticulum from long‐chain FAs, which have over 16 carbons [115]. Saturated VLCFAs contribute to insulating axons by decreasing myelin fluidity and providing a thick permeability barrier for ions. Saturated VLCFAs interact with each other through their tails (straight structure with no double bonds), thus leading to the rigidity of membranes [113]. It has been reported that a reduction in the levels of ceramide species with VLCFA residues induces myelin defects in mice [116]. Additionally, the accumulation of VLCFAs decreases myelin stability and/or synthesis of plasmalogens, which can also cause demyelination [117, 118, 119]. Another study has shown that the accumulation of VLCFAs in oligodendrocytes contributes to the loss of peroxisome function, causing demyelination, axonal degeneration, neuroinflammation, and neurodegenerative phenotypes in mice [118].

#### Fatty acid synthesis

*De novo* FA synthesis is important for the formation and growth of myelin [102, 120]. SREBP1 and SREBP cleavage activating protein (SCAP) regulate the expression of genes related to FA synthesis. SREBP1 is activated by the depletion of intracellular cholesterol; therefore, FA synthesis is associated with cholesterol synthesis [121]. The expression of FAS, induced by SREBP1, correlates with myelination in peripheral nerves during development [122]. It has also been reported that deficiency of SCAP in Schwann cells decreases the levels of saturated VLCFAs in mice [123].

#### Fatty acid transport

Fatty acids in myelinating cells are provided by endothelial cells and astrocytes by passive diffusion or by active transport through FA translocase (CD36) and FA transport proteins (FATPs) [98, 124, 125]. However, the roles of CD36 and FATPs in oligodendrocyte lineage cells and myelin have not been clarified. FATP1 is a predominant isoform expressed in the CNS, and FATP4 is also highly expressed in the brain [126]. FA‐binding proteins (FABPs) are molecular chaperones for FAs and are involved in FA transport. FABP7 and FABP5 are expressed in oligodendrocytes at different developmental stages. In mice, FABP7 has been related to the proliferation in oligodendrocytes and differentiation of immature oligodendrocytes, whereas FABP5 has been linked to differentiation of oligodendrocytes into mature myelinating oligodendrocytes. However, neither FABP7 nor FABP5 plays important roles in myelin formation [127].

#### Fatty acid oxidation

For a long time, it was considered that FA oxidation for energy requirement does not occur in oligodendrocytes because of their high demand in FAs for myelination but that it is conducted exclusively in astrocytes [128, 129]. However, some reports have suggested that the energetic profiles of myelinating cells and astrocytes are similar [98, 130, 131]. Although FA oxidation might occur in oligodendrocyte lineage cells, further analyses are needed to clarify this hypothesis.

## Abnormalities in oligodendrocytes and myelin in Alzheimer's disease brain

### Patients with Alzheimer's disease

Several studies have suggested that abnormalities of oligodendrocyte lineage cells or myelin occur in the brain of patients with AD (Fig. 2C). It has been reported that levels of several proteins linked to the oligodendrocyte lineage [myelin basic protein (MBP), myelin proteolipid protein, and cyclic nucleotide phosphohydrolase (CNPase)] are significantly lower in the white matter of postmortem AD brains. In the same study, total protein and cholesterol levels were also significantly decreased, and the composition in FAs was altered in the white matter of postmortem AD brains. These results indicate that a loss of myelin occurs in the white matter of the AD brain [132]. Olig2 is an oligodendrocyte lineage marker, and the number of Olig2‐positive cells is decreased in the brain of patients with AD [133]. Our group reported that the expression of oligodendrocyte lineage genes [*MBP*, myelin‐associated glycoprotein (*MAG*), claudin 11 (*CLDN11*), myelin oligodendrocyte glycoprotein (*MOG*), *CNP*] is decreased in the precuneus of postmortem AD brains [134]. In addition, it has been reported that the nuclear diameter of oligodendrocytes is shorter, whereas that of neurons is unchanged in the parahippocampal white matter of patients with AD [135]. A recent study using single‐cell transcriptomic analysis showed that myelination processes were recurrently perturbed in oligodendrocytes, oligodendrocyte precursor cells (OPCs), and other cell types in the prefrontal cortex of patients with AD [136].

### Alzheimer's disease animal models

Anomalies in oligodendrocyte lineage cells have also been reported in AD animal models. Mutations of PS1, a subunit of γ‐secretase, lead to a vulnerability of oligodendrocytes against toxicities induced by glutamate and Aβ peptides and are accompanied by a deficit in calcium regulation [137]. A report indicated that MBP levels and the number of myelinating oligodendrocytes are decreased in 3 x Tg AD mice. Additionally, the number of mature nonmyelinating cells was increased, whereas the number of immature oligodendrocytes remained unchanged [138]. It has also been reported that the number of OPCs was increased in APP/PS1 mice [133]. Another report showed that the proliferative rate of OPCs is increased in APP/PS1 mice. These OPCs differentiate into mature oligodendrocytes and form myelin sheaths, despite of decrease in the level of whole myelination [139, 140].

A recent study showed that myelin dysfunction in AD mice caused the accumulation of Aβ‐producing machinery within axonal swellings, and increased cleavage of APP in the cerebral cortex. Moreover, AD mice lacked microglia around plaques under the dysfunction of myelin, and similar but distinct disease‐associated microglia (DAM) signatures were induced concomitantly with myelin damage and amyloid plaques. DAM were apparently distracted by adjacent myelin damage, despite that DAM usually clear amyloid plaques [141].

These studies from patients with AD or animal models of AD suggest that, although detailed responses to AD pathology remain unclear, oligodendrocyte lineage cells and myelin are negatively affected in AD.

### Disease‐associated oligodendrocytes in Alzheimer's disease

Disease‐associated oligodendrocytes (DAOs) have been described in neurodegenerative diseases including AD. In 5xFAD mice, oligodendrocytes increased with brain pathology were termed DAOs, and expressed SERPIN3A/SERPINA3 in the diseased cortical regions and near Aβ plaques. Moreover, SERPIN3A/SERPINA3 was also expressed in the human AD brain, and this level was correlated with cognitive decline [142]. Another study indicated that three distinct DAOs were identified in mouse models of AD from single‐cell RNA sequencing: DA1 (associated with immunogenic genes), DA2 (associated with genes influencing survival), and IFN (associated with interferon response genes). DA1 and DA2 are established in the regions outside the demyelinating lesion, and DA1 repopulated in the regions with remyelination. However, the signature of oligodendrocyte activation observed in patients with AD was distinct from those observed in AD mice [143]. In App^NL‐G‐F^ mice, the activation of DAOs (Mbp^+^Cd74^+^ oligodendrocytes) was associated with the abnormality of Erk1/2 signaling. Inhibition of Erk1/2 signaling in DAOs rescued impairment of axonal myelination, and decreased Aβ pathologies and cognitive decline [144].

## Brain atrophy and white matter abnormalities in Alzheimer's disease: Human studies

### Hippocampal atrophy

Hippocampal atrophy is one of the best‐known biomarkers used in clinics for magnetic resonance imaging (MRI). Several MRI studies have indicated that hippocampal volume is reduced by 10–15% in patients with an amnestic variant of MCI and by 20–25% in the patients at the clinical AD stage [145]. Progressive rates of hippocampal atrophy are 4.66% per year for patients with AD and 1.41% per year for healthy controls [146]. A reduction in hippocampal volume for a long time correlates with cognitive decline [147, 148]. Moreover, the reduction in hippocampal volume is correlated with the severity of cognitive impairments and episodic memory deficits in patients with MCI and AD [149]. On the contrary, hippocampal atrophy is also observed in patients with vascular dementia [150, 151], semantic dementia [152], Parkinson's disease dementia [151, 153], and frontotemporal lobar degeneration [154]. Moreover, hippocampal volume correlates with Braak staging and the remaining number of neurons in dementia and aging [153, 155, 156, 157].

### White matter abnormalities

White matter abnormalities are observed in cerebrovascular diseases. However, recently, it has been considered a hallmark of AD. White matter hyperintensities (WMH) are signal anomalies visualized by T2‐weighted MRI. WMH are especially observed in deep periventricular white matter, where the blood perfusion rate is low. The blood flow in the white matter is reduced by aging and AD, resulting in hypoxic and ischemic damage and lower vessel densities [158, 159]. Nasrabady and colleagues reported that WMH predicted the incidence of AD [160, 161, 162] and the degree of cognitive impairment in patients with AD [163]. They also indicated that WMH were associated with the *APOE4* risk genes in late‐onset AD [164]. Recently, a study from Dominantly Inherited Alzheimer's Network (DIAN) showed that WMH volume is increased in patients with autosomal dominant and fully penetrant AD mutations, as early as 20 years before the expected onset of symptoms. In these preclinical patients, WMH severity correlates with Aβ1–42 levels in CSF [165]. Notably, the association between WMH severity and amyloid levels in CSF was independent of vascular risk factors [166]. Additionally, an increase in tau levels in CSF is predicted by WMH severity in patients with MCI [167, 168]. On the contrary, vascular and BBB impairments, small hemorrhagic lesions, and iron accumulation have been observed in the brain of patients with preclinical AD [169]. However, neuroimaging studies have shown that white matter networks are already defective at the preclinical AD stage in the absence of neurodegenerative changes, cortical atrophy, or cortical glucose reduction [170]. Studies in AD animal models also suggest that white matter defects are observed before the appearance of cortical amyloid plaques and tangles [138, 171]. McAleese and colleagues evaluated the effect of neurodegeneration on white matter abnormalities in the parietal and frontal cortex of AD brains. In the parietal cortex, WMH pathogenesis associated with AD was linked to Wallerian‐like degeneration caused by AD pathology, whereas WMH occurring without AD were related to vasculopathy and ischemia. In contrast, in the frontal cortex, WMH pathogenesis associated with AD was related to both degenerative and vasculopathy mechanisms [172, 173]. It has been speculated that neurodegeneration in AD induces white matter abnormalities in specific regions of the brain.

### White matter abnormalities and myelin

Myeline sheaths are abundant in the white matter. WMH have been histopathologically associated with myelin pallor, loss of myelin, and loss of myelinated axons, which are accompanied by changes in the arterial adventitia in deep white matter [174, 175, 176]. A loss of myelin was observed in the AD brains, particularly in the forebrain, entorhinal cortex, hippocampus, and amygdala (regions myelinated later during normal development of the CNS). The loss of myelin in these regions was significantly greater than that in the spinal cord and brain stem (regions myelinated earlier during normal development of the CNS) [177, 178, 179, 180, 181]. It is suggested that a loss of myelin participates in white matter abnormalities, which may exacerbate brain atrophy in AD.

## Association between obesity and Alzheimer's disease

Obesity is a known risk factor for AD. Several epidemiological studies have indicated that the risk of developing dementia is increased by obesity in midlife (age in 50s and 60s). Some of these reports showed an increased risk of AD in obese humans, particularly in women [182, 183, 184, 185, 186]. A study focusing on the alteration of lipid compositions in the brain of obese APP23 mice fed a high‐fat diet (HFD) was conducted using lipidome analysis. Although the total amount of phospholipids was not changed, the levels of 24 lipid species were significantly altered by the HFD. Particularly, the analysis of network visualization of correlated lipids revealed that HFD induced an overall imbalance, with the most remarkable effect being on cardiolipin molecular subspecies [187]. On the contrary, epidemiological studies indicated that obesity in late life (≧ 60 years old) was not associated with a higher risk for earlier onset of AD [182, 188]. In addition, obese humans often have other metabolic disorders, such as type 2 diabetes, hypercholesterolemia, and hypertension, which can induce cardiovascular diseases and have been associated with dementia and AD [9].

### Impacts of obesity on brain functions in rodents fed a high‐fat diet

The impacts of obesity on brain functions are widely studied using rodents fed an HFD [189]. In mice, HFD increases the levels of Aβ in the hippocampus and induces amyloid depositions in the brain [190, 191]. Furthermore, several studies using mice and rats showed that neuroinflammation was induced by HFD. HFD increases the expression levels of nuclear factor‐κB, interleukin‐1β, and toll‐like receptor 4, and densities of astrocytes and microglia in the brain [191, 192, 193]. Neuroinflammation induced by HFD has been related to BBB leakage [189, 194, 195]. In addition, several studies have indicated that rodents fed HFD exhibited impaired working [196], spatial [197], sustained recognition [193], long‐term [198], and episodic [199] memories. These memory impairments induced by HFD have been associated with synaptic dysfunctions and neuronal death, synaptic degeneration in the hippocampus and cerebral cortex [200], an increase in apoptotic signals in the hippocampus [191], and a decrease in acetylcholinesterase activity in various regions including the prefrontal cortex [201]. On the contrary, in APP23‐ob/ob mice, the AD mouse model crossed with the obese mice by overeating of a normal diet, Aβ burden was not increased, and the expression of microglial markers were down‐regulated in the brain. However, learning and memory deficit were exacerbated in these mice [202, 203]. Further analyses are needed to clarify the association between obesity and the AD pathologies.

### Obesity and brain atrophy in humans

Obesity has been associated with brain atrophy [204]. Studies using diffusion‐weighted imaging (diffusion MRI) have indicated that obesity measures are negatively correlated with fiber connectivity [205, 206, 207, 208, 209, 210]. White matter structure in the corpus callosum (genu, trunk, and splenium), cerebellar peduncle, corona radiata [209, 210], fornix [210], and uncinate fasciculus [206] is altered in obese older adults. White matter volume in obese humans has also been related to a greater degree of atrophy, which is maximal in middle age (approximately 40 years old) and corresponds to an estimated increase in brain age by 10 years [211]. On the contrary, other studies have not found any association between obesity and white matter integrity or positive interaction between body mass index and white matter integrity and volume [212, 213, 214]. In addition, the reduction in lean body mass in patients with early‐stage AD has been related to brain atrophy and lower cognitive performance, when controlling for age and sex [215]. Thus, although obesity seems to be associated with brain atrophy and white matter integrity, more analyses are required.

## Effects of dietary intake of specific lipid species on Alzheimer's disease

Regardless of obesity, dietary intake of several lipid species and internal lipid conditions may affect AD pathology. Epidemiological studies have indicated that AD risk is decreased by high dietary intake of ω‐3 PUFAs [especially DHA and eicosapentaenoic acid (EPA)] and is increased by low intake of these ω‐3 PUFAs [216, 217]. Omega‐3 PUFAs are involved in inflammation response against Aβ [218] and activate RXR and PPARs, which directly affect Aβ metabolism [219, 220, 221]. DHA and EPA contribute to reducing Aβ levels by lowering the activity of β‐ and γ‐secretases and by stimulating insulin‐degrading enzyme, which degrades Aβ [222, 223]. On the contrary, randomized clinical trials showed that dietary supplementation with DHA did not delay cognitive decline in patients with mild‐to‐moderate AD. Subgroup analysis in these trials showed a positive effect of DHA in ApoE4 noncarrier patients with very mild AD [224]. The effect of a ketogenic diet (comprising high levels of saturated fats and low amounts of carbohydrates) has also been assessed in patients with AD and animal models of AD. Ketogenic diet reduces Aβ pathology and improves metabolic and cognitive functions in both aging and AD animal models [225, 226]. Although ketogenic interventions were effective in early‐phase clinical trials, further studies showing long‐term effects on AD are required [9, 227, 228, 229, 230].

Diets with specific FA composition have been linked to the development or maintenance of myelin. Diets excluding essential FAs lead to alterations in FA composition of myelin and cause myelin splitting, but do not significantly affect myelination [231]. A ketogenic diet has been reported to reduce axonal degeneration and improve motor functions in a mouse model of Pelizaeus–Merzbacher disease [232].

## Conclusions and perspectives

**Fig. 3:**
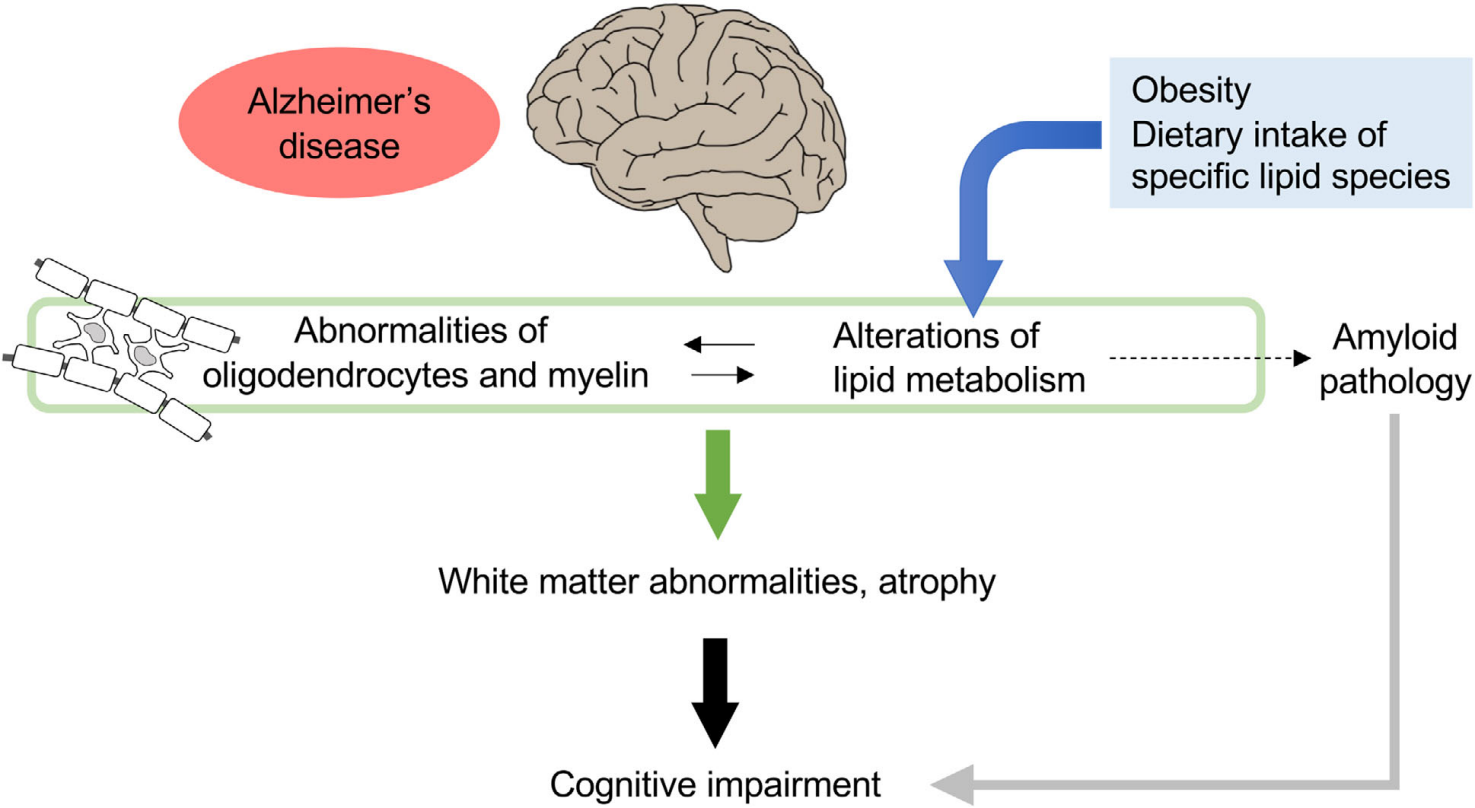
Hypothetical mechanism showing that alterations in lipid metabolism in the brain exacerbate AD pathology by inducing oligodendrocyte abnormalities. Lipid metabolism may be altered in the AD brain, particularly in myelin or oligodendrocyte lineage cells, which may cause white matter abnormalities and/or atrophy leading to cognitive impairment. Moreover, obesity and diets containing specific lipids might exacerbate or improve AD pathology.

The lipid composition of the brain is altered in AD, and several lipid species may affect the functions of oligodendrocytes and myelin. Therefore, lipid metabolism is likely altered in the AD brain, particularly in myelin or oligodendrocyte lineage cells, which are vulnerable to lipidic changes. In addition, several studies have shown white matter abnormalities and oligodendrocyte dysfunctions in AD brains. Further analyses are required to define the association between the alteration of brain lipid metabolism and dysfunction of oligodendrocytes and white matter abnormalities in the AD brain. Moreover, obesity is a risk factor for AD, and several studies have shown a link between obesity and brain atrophy. Dietary intake of specific lipids may improve AD pathology. In the future, the contribution of peripheral dietary intake to AD pathology needs to be clarified (Fig. 3).

## Author contribution

N. K. and K. Y. wrote the manuscript, and prepared the figures.

## Conflict of interest

The authors declare no conflict of interest.

## References

[1] Yoon JH , Seo Y , Jo YS , Lee S , Cho E , Cazenave‐Gassiot A , Shin YS , Moon MH , An HJ , Wenk MR *et al*. (2022) Brain lipidomics: from functional landscape to clinical significance. Sci Adv 8, eadc9317. 36112688 10.1126/sciadv.adc9317 PMC9481132

[2] Kao YC , Ho PC , Tu YK , Jou IM and Tsai KJ (2020) Lipids and Alzheimer's disease. Int J Mol Sci 21, 1505. 32098382 10.3390/ijms21041505 PMC7073164

[3] Tracey TJ , Steyn FJ , Wolvetang EJ and Ngo ST (2018) Neuronal lipid metabolism: multiple pathways driving functional outcomes in health and disease. Front Mol Neurosci 11, 10. 29410613 10.3389/fnmol.2018.00010 PMC5787076

[4] Pifferi F , Laurent B and Plourde M (2021) Lipid transport and metabolism at the blood‐brain Interface: implications in health and disease. Front Physiol 12, 645646. 33868013 10.3389/fphys.2021.645646 PMC8044814

[5] Alessandri JM , Extier A , Al‐Gubory KH , Langelier B , Baudry C , LePoupon C , Lavialle M and Guesnet P (2011) Ovariectomy and 17beta‐estradiol alter transcription of lipid metabolism genes and proportions of neo‐formed n‐3 and n‐6 long‐chain polyunsaturated fatty acids differently in brain and liver. J Nutr Biochem 22, 820–827. 21129945 10.1016/j.jnutbio.2010.07.005

[6] Dyall SC , Balas L , Bazan NG , Brenna JT , Chiang N , da Costa Souza F , Dalli J , Durand T , Galano JM , Lein PJ *et al*. (2022) Polyunsaturated fatty acids and fatty acid‐derived lipid mediators: recent advances in the understanding of their biosynthesis, structures, and functions. Prog Lipid Res 86, 101165. 35508275 10.1016/j.plipres.2022.101165 PMC9346631

[7] Bazinet RP and Laye S (2014) Polyunsaturated fatty acids and their metabolites in brain function and disease. Nat Rev Neurosci 15, 771–785. 25387473 10.1038/nrn3820

[8] Ebert D , Haller RG and Walton ME (2003) Energy contribution of octanoate to intact rat brain metabolism measured by 13C nuclear magnetic resonance spectroscopy. J Neurosci 23, 5928–5935. 12843297 10.1523/JNEUROSCI.23-13-05928.2003 PMC6741266

[9] Yin F (2023) Lipid metabolism and Alzheimer's disease: clinical evidence, mechanistic link and therapeutic promise. FEBS J 290, 1420–1453. 34997690 10.1111/febs.16344 PMC9259766

[10] Naudi A , Cabre R , Jove M , Ayala V , Gonzalo H , Portero‐Otin M , Ferrer I and Pamplona R (2015) Lipidomics of human brain aging and Alzheimer's disease pathology. Int Rev Neurobiol 122, 133–189. 26358893 10.1016/bs.irn.2015.05.008

[11] Farooqui AA , Horrocks LA and Farooqui T (2000) Glycerophospholipids in brain: their metabolism, incorporation into membranes, functions, and involvement in neurological disorders. Chem Phys Lipids 106, 1–29. 10878232 10.1016/s0009-3084(00)00128-6

[12] Dennis EA , Rhee SG , Billah MM and Hannun YA (1991) Role of phospholipase in generating lipid second messengers in signal transduction. FASEB J 5, 2068–2077. 1901288 10.1096/fasebj.5.7.1901288

[13] Hussain G , Wang J , Rasul A , Anwar H , Imran A , Qasim M , Zafar S , Kamran SKS , Razzaq A , Aziz N *et al*. (2019) Role of cholesterol and sphingolipids in brain development and neurological diseases. Lipids Health Dis 18, 26. 30683111 10.1186/s12944-019-0965-z PMC6347843

[14] Olsen ASB and Faergeman NJ (2017) Sphingolipids: membrane microdomains in brain development, function and neurological diseases. Open Biol 7, 170069. 28566300 10.1098/rsob.170069 PMC5451547

[15] Fantini J and Barrantes FJ (2009) Sphingolipid/cholesterol regulation of neurotransmitter receptor conformation and function. Biochim Biophys Acta 1788, 2345–2361. 19733149 10.1016/j.bbamem.2009.08.016

[16] Arenas F , Garcia‐Ruiz C and Fernandez‐Checa JC (2017) Intracellular cholesterol trafficking and impact in neurodegeneration. Front Mol Neurosci 10, 382. 29204109 10.3389/fnmol.2017.00382 PMC5698305

[17] Zhang J and Liu Q (2015) Cholesterol metabolism and homeostasis in the brain. Protein Cell 6, 254–264. 25682154 10.1007/s13238-014-0131-3 PMC4383754

[18] Lund EG , Xie C , Kotti T , Turley SD , Dietschy JM and Russell DW (2003) Knockout of the cholesterol 24‐hydroxylase gene in mice reveals a brain‐specific mechanism of cholesterol turnover. J Biol Chem 278, 22980–22988. 12686551 10.1074/jbc.M303415200

[19] Bjorkhem I , Cedazo‐Minguez A , Leoni V and Meaney S (2009) Oxysterols and neurodegenerative diseases. Mol Aspects Med 30, 171–179. 19248803 10.1016/j.mam.2009.02.001

[20] Gamba P , Giannelli S , Staurenghi E , Testa G , Sottero B , Biasi F , Poli G and Leonarduzzi G (2021) The controversial role of 24‐S‐hydroxycholesterol in Alzheimer's disease. Antioxidants 10, 740. 34067119 10.3390/antiox10050740 PMC8151638

[21] Bjorkhem I (2006) Crossing the barrier: oxysterols as cholesterol transporters and metabolic modulators in the brain. J Intern Med 260, 493–508. 17116000 10.1111/j.1365-2796.2006.01725.x

[22] Iuliano L , Crick PJ , Zerbinati C , Tritapepe L , Abdel‐Khalik J , Poirot M , Wang Y and Griffiths WJ (2015) Cholesterol metabolites exported from human brain. Steroids 99, 189–193. 25668615 10.1016/j.steroids.2015.01.026 PMC4503873

[23] Meaney S , Heverin M , Panzenboeck U , Ekstrom L , Axelsson M , Andersson U , Diczfalusy U , Pikuleva I , Wahren J , Sattler W *et al*. (2007) Novel route for elimination of brain oxysterols across the blood‐brain barrier: conversion into 7alpha‐hydroxy‐3‐oxo‐4‐cholestenoic acid. J Lipid Res 48, 944–951. 17251592 10.1194/jlr.M600529-JLR200

[24] Marwarha G and Ghribi O (2015) Does the oxysterol 27‐hydroxycholesterol underlie Alzheimer's disease‐Parkinson's disease overlap? Exp Gerontol 68, 13–18. 25261765 10.1016/j.exger.2014.09.013 PMC4377123

[25] Heverin M , Bogdanovic N , Lutjohann D , Bayer T , Pikuleva I , Bretillon L , Diczfalusy U , Winblad B and Bjorkhem I (2004) Changes in the levels of cerebral and extracerebral sterols in the brain of patients with Alzheimer's disease. J Lipid Res 45, 186–193. 14523054 10.1194/jlr.M300320-JLR200

[26] Lund EG , Kerr TA , Sakai J , Li WP and Russell DW (1998) cDNA cloning of mouse and human cholesterol 25‐hydroxylases, polytopic membrane proteins that synthesize a potent oxysterol regulator of lipid metabolism. J Biol Chem 273, 34316–34327. 9852097 10.1074/jbc.273.51.34316

[27] Madenspacher JH , Morrell ED , Gowdy KM , McDonald JG , Thompson BM , Muse G , Martinez J , Thomas S , Mikacenic C , Nick JA *et al*. (2020) Cholesterol 25‐hydroxylase promotes efferocytosis and resolution of lung inflammation. JCI Insight 5, e137189. 32343675 10.1172/jci.insight.137189 PMC7308063

[28] Cyster JG , Dang EV , Reboldi A and Yi T (2014) 25‐hydroxycholesterols in innate and adaptive immunity. Nat Rev Immunol 14, 731–743. 25324126 10.1038/nri3755

[29] Wong MY , Lewis M , Doherty JJ , Shi Y , Cashikar AG , Amelianchik A , Tymchuk S , Sullivan PM , Qian M , Covey DF *et al*. (2020) 25‐hydroxycholesterol amplifies microglial IL‐1beta production in an apoE isoform‐dependent manner. J Neuroinflammation 17, 192. 32552741 10.1186/s12974-020-01869-3 PMC7298825

[30] Odnoshivkina UG , Kuznetsova EA and Petrov AM (2022) 25‐hydroxycholesterol as a signaling molecule of the nervous system. Biochemistry 87, 524–537. 35790411 10.1134/S0006297922060049 PMC9201265

[31] Janssen CI and Kiliaan AJ (2014) Long‐chain polyunsaturated fatty acids (LCPUFA) from genesis to senescence: the influence of LCPUFA on neural development, aging, and neurodegeneration. Prog Lipid Res 53, 1–17. 24334113 10.1016/j.plipres.2013.10.002

[32] Cunnane SC , Schneider JA , Tangney C , Tremblay‐Mercier J , Fortier M , Bennett DA and Morris MC (2012) Plasma and brain fatty acid profiles in mild cognitive impairment and Alzheimer's disease. J Alzheimers Dis 29, 691–697. 22466064 10.3233/JAD-2012-110629 PMC3409580

[33] Snowden SG , Ebshiana AA , Hye A , An Y , Pletnikova O , O'Brien R , Troncoso J , Legido‐Quigley C and Thambisetty M (2017) Association between fatty acid metabolism in the brain and Alzheimer disease neuropathology and cognitive performance: a nontargeted metabolomic study. PLoS Med 14, e1002266. 28323825 10.1371/journal.pmed.1002266 PMC5360226

[34] Martin V , Fabelo N , Santpere G , Puig B , Marin R , Ferrer I and Diaz M (2010) Lipid alterations in lipid rafts from Alzheimer's disease human brain cortex. J Alzheimers Dis 19, 489–502. 20110596 10.3233/JAD-2010-1242

[35] Fabelo N , Martin V , Marin R , Moreno D , Ferrer I and Diaz M (2014) Altered lipid composition in cortical lipid rafts occurs at early stages of sporadic Alzheimer's disease and facilitates APP/BACE1 interactions. Neurobiol Aging 35, 1801–1812. 24613671 10.1016/j.neurobiolaging.2014.02.005

[36] Prasad MR , Lovell MA , Yatin M , Dhillon H and Markesbery WR (1998) Regional membrane phospholipid alterations in Alzheimer's disease. Neurochem Res 23, 81–88. 9482271 10.1023/a:1022457605436

[37] Belkouch M , Hachem M , Elgot A , Lo Van A , Picq M , Guichardant M , Lagarde M and Bernoud‐Hubac N (2016) The pleiotropic effects of omega‐3 docosahexaenoic acid on the hallmarks of Alzheimer's disease. J Nutr Biochem 38, 1–11. 27825512 10.1016/j.jnutbio.2016.03.002

[38] Fonteh AN , Cipolla M , Chiang AJ , Edminster SP , Arakaki X and Harrington MG (2020) Polyunsaturated fatty acid composition of cerebrospinal fluid fractions shows their contribution to cognitive resilience of a pre‐symptomatic Alzheimer's disease cohort. Front Physiol 11, 83. 32116789 10.3389/fphys.2020.00083 PMC7034243

[39] Fonteh AN , Cipolla M , Chiang J , Arakaki X and Harrington MG (2014) Human cerebrospinal fluid fatty acid levels differ between supernatant fluid and brain‐derived nanoparticle fractions, and are altered in Alzheimer's disease. PLoS One 9, e100519. 24956173 10.1371/journal.pone.0100519 PMC4067345

[40] Guan Z , Wang Y , Cairns NJ , Lantos PL , Dallner G and Sindelar PJ (1999) Decrease and structural modifications of phosphatidylethanolamine plasmalogen in the brain with Alzheimer disease. J Neuropathol Exp Neurol 58, 740–747. 10411344 10.1097/00005072-199907000-00008

[41] Nitsch RM , Blusztajn JK , Pittas AG , Slack BE , Growdon JH and Wurtman RJ (1992) Evidence for a membrane defect in Alzheimer disease brain. Proc Natl Acad Sci U S A 89, 1671–1675. 1311847 10.1073/pnas.89.5.1671 PMC48514

[42] Pettegrew JW , Panchalingam K , Hamilton RL and McClure RJ (2001) Brain membrane phospholipid alterations in Alzheimer's disease. Neurochem Res 26, 771–782. 11565608 10.1023/a:1011603916962

[43] Stokes CE and Hawthorne JN (1987) Reduced phosphoinositide concentrations in anterior temporal cortex of Alzheimer‐diseased brains. J Neurochem 48, 1018–1021. 3029323 10.1111/j.1471-4159.1987.tb05619.x

[44] Han X , Holtzman DM and McKeel DW Jr (2001) Plasmalogen deficiency in early Alzheimer's disease subjects and in animal models: molecular characterization using electrospray ionization mass spectrometry. J Neurochem 77, 1168–1180. 11359882 10.1046/j.1471-4159.2001.00332.x

[45] Cutler RG , Kelly J , Storie K , Pedersen WA , Tammara A , Hatanpaa K , Troncoso JC and Mattson MP (2004) Involvement of oxidative stress‐induced abnormalities in ceramide and cholesterol metabolism in brain aging and Alzheimer's disease. Proc Natl Acad Sci U S A 101, 2070–2075. 14970312 10.1073/pnas.0305799101 PMC357053

[46] Filippov V , Song MA , Zhang K , Vinters HV , Tung S , Kirsch WM , Yang J and Duerksen‐Hughes PJ (2012) Increased ceramide in brains with Alzheimer's and other neurodegenerative diseases. J Alzheimers Dis 29, 537–547. 22258513 10.3233/JAD-2011-111202 PMC3643694

[47] Han X , Holtzman DM , McKeel DW Jr , Kelley J and Morris JC (2002) Substantial sulfatide deficiency and ceramide elevation in very early Alzheimer's disease: potential role in disease pathogenesis. J Neurochem 82, 809–818. 12358786 10.1046/j.1471-4159.2002.00997.x

[48] He X , Huang Y , Li B , Gong CX and Schuchman EH (2010) Deregulation of sphingolipid metabolism in Alzheimer's disease. Neurobiol Aging 31, 398–408. 18547682 10.1016/j.neurobiolaging.2008.05.010 PMC2829762

[49] Kosicek M , Zetterberg H , Andreasen N , Peter‐Katalinic J and Hecimovic S (2012) Elevated cerebrospinal fluid sphingomyelin levels in prodromal Alzheimer's disease. Neurosci Lett 516, 302–305. 22521584 10.1016/j.neulet.2012.04.019

[50] Soderberg M , Edlund C , Alafuzoff I , Kristensson K and Dallner G (1992) Lipid composition in different regions of the brain in Alzheimer's disease/senile dementia of Alzheimer's type. J Neurochem 59, 1646–1653. 1402910 10.1111/j.1471-4159.1992.tb10994.x

[51] Couttas TA , Kain N , Suchowerska AK , Quek LE , Turner N , Fath T , Garner B and Don AS (2016) Loss of ceramide synthase 2 activity, necessary for myelin biosynthesis, precedes tau pathology in the cortical pathogenesis of Alzheimer's disease. Neurobiol Aging 43, 89–100. 27255818 10.1016/j.neurobiolaging.2016.03.027

[52] Cheng H , Wang M , Li JL , Cairns NJ and Han X (2013) Specific changes of sulfatide levels in individuals with pre‐clinical Alzheimer's disease: an early event in disease pathogenesis. J Neurochem 127, 733–738. 23865640 10.1111/jnc.12368 PMC3844035

[53] Liu Y , Zhong X , Shen J , Jiao L , Tong J , Zhao W , Du K , Gong S , Liu M and Wei M (2020) Elevated serum TC and LDL‐C levels in Alzheimer's disease and mild cognitive impairment: a meta‐analysis study. Brain Res 1727, 146554. 31765631 10.1016/j.brainres.2019.146554

[54] Popp J , Meichsner S , Kolsch H , Lewczuk P , Maier W , Kornhuber J , Jessen F and Lutjohann D (2013) Cerebral and extracerebral cholesterol metabolism and CSF markers of Alzheimer's disease. Biochem Pharmacol 86, 37–42. 23291240 10.1016/j.bcp.2012.12.007

[55] Mori T , Paris D , Town T , Rojiani AM , Sparks DL , Delledonne A , Crawford F , Abdullah LI , Humphrey JA , Dickson DW *et al*. (2001) Cholesterol accumulates in senile plaques of Alzheimer disease patients and in transgenic APP(SW) mice. J Neuropathol Exp Neurol 60, 778–785. 11487052 10.1093/jnen/60.8.778

[56] Chan RB , Oliveira TG , Cortes EP , Honig LS , Duff KE , Small SA , Wenk MR , Shui G and Di Paolo G (2012) Comparative lipidomic analysis of mouse and human brain with Alzheimer disease. J Biol Chem 287, 2678–2688. 22134919 10.1074/jbc.M111.274142 PMC3268426

[57] Tajima Y , Ishikawa M , Maekawa K , Murayama M , Senoo Y , Nishimaki‐Mogami T , Nakanishi H , Ikeda K , Arita M , Taguchi R *et al*. (2013) Lipidomic analysis of brain tissues and plasma in a mouse model expressing mutated human amyloid precursor protein/tau for Alzheimer's disease. Lipids Health Dis 12, 68. 23659495 10.1186/1476-511X-12-68 PMC3668217

[58] Hascalovici JR , Vaya J , Khatib S , Holcroft CA , Zukor H , Song W , Arvanitakis Z , Bennett DA and Schipper HM (2009) Brain sterol dysregulation in sporadic AD and MCI: relationship to heme oxygenase‐1. J Neurochem 110, 1241–1253. 19522732 10.1111/j.1471-4159.2009.06213.x PMC2770254

[59] Varma VR , Busra Luleci H , Oommen AM , Varma S , Blackshear CT , Griswold ME , An Y , Roberts JA , O'Brien R , Pletnikova O *et al*. (2021) Abnormal brain cholesterol homeostasis in Alzheimer's disease‐a targeted metabolomic and transcriptomic study. NPJ Aging Mech Dis 7, 11. 34075056 10.1038/s41514-021-00064-9 PMC8169871

[60] Testa G , Staurenghi E , Zerbinati C , Gargiulo S , Iuliano L , Giaccone G , Fanto F , Poli G , Leonarduzzi G and Gamba P (2016) Changes in brain oxysterols at different stages of Alzheimer's disease: their involvement in neuroinflammation. Redox Biol 10, 24–33. 27687218 10.1016/j.redox.2016.09.001 PMC5040635

[61] Blalock EM , Buechel HM , Popovic J , Geddes JW and Landfield PW (2011) Microarray analyses of laser‐captured hippocampus reveal distinct gray and white matter signatures associated with incipient Alzheimer's disease. J Chem Neuroanat 42, 118–126. 21756998 10.1016/j.jchemneu.2011.06.007 PMC3163806

[62] Holtman IR , Raj DD , Miller JA , Schaafsma W , Yin Z , Brouwer N , Wes PD , Moller T , Orre M , Kamphuis W *et al*. (2015) Induction of a common microglia gene expression signature by aging and neurodegenerative conditions: a co‐expression meta‐analysis. Acta Neuropathol Commun 3, 31. 26001565 10.1186/s40478-015-0203-5 PMC4489356

[63] Matarin M , Salih DA , Yasvoina M , Cummings DM , Guelfi S , Liu W , Nahaboo Solim MA , Moens TG , Paublete RM , Ali SS *et al*. (2015) A genome‐wide gene‐expression analysis and database in transgenic mice during development of amyloid or tau pathology. Cell Rep 10, 633–644. 25620700 10.1016/j.celrep.2014.12.041

[64] Orre M , Kamphuis W , Osborn LM , Jansen AHP , Kooijman L , Bossers K and Hol EM (2014) Isolation of glia from Alzheimer's mice reveals inflammation and dysfunction. Neurobiol Aging 35, 2746–2760. 25002035 10.1016/j.neurobiolaging.2014.06.004

[65] Wood PL , Barnette BL , Kaye JA , Quinn JF and Woltjer RL (2015) Non‐targeted lipidomics of CSF and frontal cortex grey and white matter in control, mild cognitive impairment, and Alzheimer's disease subjects. Acta Neuropsychiatr 27, 270–278. 25858158 10.1017/neu.2015.18

[66] Wood PL , Medicherla S , Sheikh N , Terry B , Phillipps A , Kaye JA , Quinn JF and Woltjer RL (2015) Targeted Lipidomics of fontal cortex and plasma diacylglycerols (DAG) in mild cognitive impairment and Alzheimer's disease: validation of DAG accumulation early in the pathophysiology of Alzheimer's disease. J Alzheimers Dis 48, 537–546. 26402017 10.3233/JAD-150336 PMC4713833

[67] Zhang X , Liu W , Cao Y and Tan W (2020) Hippocampus proteomics and brain Lipidomics reveal network dysfunction and lipid molecular abnormalities in APP/PS1 mouse model of Alzheimer's disease. J Proteome Res 19, 3427–3437. 32510958 10.1021/acs.jproteome.0c00255

[68] Zhang X , Liu W , Zan J , Wu C and Tan W (2020) Untargeted lipidomics reveals progression of early Alzheimer's disease in APP/PS1 transgenic mice. Sci Rep 10, 14509. 32884056 10.1038/s41598-020-71510-z PMC7471266

[69] Emre C , Do KV , Jun B , Hjorth E , Alcalde SG , Kautzmann MI , Gordon WC , Nilsson P , Bazan NG and Schultzberg M (2021) Age‐related changes in brain phospholipids and bioactive lipids in the APP knock‐in mouse model of Alzheimer's disease. Acta Neuropathol Commun 9, 116. 34187579 10.1186/s40478-021-01216-4 PMC8244172

[70] Bandaru VV , Troncoso J , Wheeler D , Pletnikova O , Wang J , Conant K and Haughey NJ (2009) ApoE4 disrupts sterol and sphingolipid metabolism in Alzheimer's but not normal brain. Neurobiol Aging 30, 591–599. 17888544 10.1016/j.neurobiolaging.2007.07.024 PMC2758772

[71] Lane RM and Farlow MR (2005) Lipid homeostasis and apolipoprotein E in the development and progression of Alzheimer's disease. J Lipid Res 46, 949–968. 15716586 10.1194/jlr.M400486-JLR200

[72] Kline A (2012) Apolipoprotein E, amyloid‐ß clearance and therapeutic opportunities in Alzheimer's disease. Alzheimers Res Ther 4, 32. 22929359 10.1186/alzrt135 PMC3506946

[73] Nunes VS , Cazita PM , Catanozi S , Nakandakare ER and Quintao ECR (2018) Decreased content, rate of synthesis and export of cholesterol in the brain of apoE knockout mice. J Bioenerg Biomembr 50, 283–287. 29675736 10.1007/s10863-018-9757-9

[74] Fuentes D , Fernandez N , Garcia Y , Garcia T , Morales AR and Menendez R (2018) Age‐related changes in the behavior of apolipoprotein E knockout mice. Behav Sci 8, 33. 29510495 10.3390/bs8030033 PMC5867486

[75] Gouna G , Klose C , Bosch‐Queralt M , Liu L , Gokce O , Schifferer M , Cantuti‐Castelvetri L and Simons M (2021) TREM2‐dependent lipid droplet biogenesis in phagocytes is required for remyelination. J Exp Med 218, e20210227. 34424266 10.1084/jem.20210227 PMC8404472

[76] Nugent AA , Lin K , van Lengerich B , Lianoglou S , Przybyla L , Davis SS , Llapashtica C , Wang J , Kim DJ , Xia D *et al*. (2020) TREM2 regulates microglial cholesterol metabolism upon chronic phagocytic challenge. Neuron 105, 837–854.e9. 31902528 10.1016/j.neuron.2019.12.007

[77] Barbero‐Camps E , Fernandez A , Martinez L , Fernandez‐Checa JC and Colell A (2013) APP/PS1 mice overexpressing SREBP‐2 exhibit combined Aβ accumulation and tau pathology underlying Alzheimer's disease. Hum Mol Genet 22, 3460–3476. 23648430 10.1093/hmg/ddt201 PMC3736868

[78] Wang H , Kulas JA , Wang C , Holtzman DM , Ferris HA and Hansen SB (2021) Regulation of beta‐amyloid production in neurons by astrocyte‐derived cholesterol. Proc Natl Acad Sci U S A 118, e2102191118. 34385305 10.1073/pnas.2102191118 PMC8379952

[79] Ates G , Goldberg J , Currais A and Maher P (2020) CMS121, a fatty acid synthase inhibitor, protects against excess lipid peroxidation and inflammation and alleviates cognitive loss in a transgenic mouse model of Alzheimer's disease. Redox Biol 36, 101648. 32863221 10.1016/j.redox.2020.101648 PMC7394765

[80] Thangavel R , Kempuraj D , Zaheer S , Raikwar S , Ahmed ME , Selvakumar GP , Iyer SS and Zaheer A (2017) Glia maturation factor and mitochondrial uncoupling proteins 2 and 4 expression in the temporal cortex of Alzheimer's disease brain. Front Aging Neurosci 9, 150. 28572767 10.3389/fnagi.2017.00150 PMC5435744

[81] Daugherty D , Goldberg J , Fischer W , Dargusch R , Maher P and Schubert D (2017) A novel Alzheimer's disease drug candidate targeting inflammation and fatty acid metabolism. Alzheimers Res Ther 9, 50. 28709449 10.1186/s13195-017-0277-3 PMC5513091

[82] de la Monte SM and Wands JR (2006) Molecular indices of oxidative stress and mitochondrial dysfunction occur early and often progress with severity of Alzheimer's disease. J Alzheimers Dis 9, 167–181. 16873964 10.3233/jad-2006-9209

[83] Wanders RJ (2004) Peroxisomes, lipid metabolism, and peroxisomal disorders. Mol Genet Metab 83, 16–27. 15464416 10.1016/j.ymgme.2004.08.016

[84] Kou J , Kovacs GG , Hoftberger R , Kulik W , Brodde A , Forss‐Petter S , Honigschnabl S , Gleiss A , Brugger B , Wanders R *et al*. (2011) Peroxisomal alterations in Alzheimer's disease. Acta Neuropathol 122, 271–283. 21594711 10.1007/s00401-011-0836-9 PMC3168371

[85] Hamilton LK , Dufresne M , Joppe SE , Petryszyn S , Aumont A , Calon F , Barnabe‐Heider F , Furtos A , Parent M , Chaurand P *et al*. (2015) Aberrant lipid metabolism in the forebrain niche suppresses adult neural stem cell proliferation in an animal model of Alzheimer's disease. Cell Stem Cell 17, 397–411. 26321199 10.1016/j.stem.2015.08.001

[86] Tong Y , Sun Y , Tian X , Zhou T , Wang H , Zhang T , Zhan R , Zhao L , Kuerban B , Li Z *et al*. (2015) Phospholipid transfer protein (PLTP) deficiency accelerates memory dysfunction through altering amyloid precursor protein (APP) processing in a mouse model of Alzheimer's disease. Hum Mol Genet 24, 5388–5403. 26160914 10.1093/hmg/ddv262

[87] Landman N , Jeong SY , Shin SY , Voronov SV , Serban G , Kang MS , Park MK , Di Paolo G , Chung S and Kim TW (2006) Presenilin mutations linked to familial Alzheimer's disease cause an imbalance in phosphatidylinositol 4,5‐bisphosphate metabolism. Proc Natl Acad Sci U S A 103, 19524–19529. 17158800 10.1073/pnas.0604954103 PMC1748258

[88] Di Paolo G and Kim TW (2011) Linking lipids to Alzheimer's disease: cholesterol and beyond. Nat Rev Neurosci 12, 284–296. 21448224 10.1038/nrn3012 PMC3321383

[89] Grimm MO , Grimm HS , Patzold AJ , Zinser EG , Halonen R , Duering M , Tschape JA , De Strooper B , Muller U , Shen J *et al*. (2005) Regulation of cholesterol and sphingomyelin metabolism by amyloid‐beta and presenilin. Nat Cell Biol 7, 1118–1123. 16227967 10.1038/ncb1313

[90] Zha Q , Ruan Y , Hartmann T , Beyreuther K and Zhang D (2004) GM1 ganglioside regulates the proteolysis of amyloid precursor protein. Mol Psychiatry 9, 946–952. 15052275 10.1038/sj.mp.4001509

[91] Hong S , Ostaszewski BL , Yang T , O'Malley TT , Jin M , Yanagisawa K , Li S , Bartels T and Selkoe DJ (2014) Soluble β oligomers are rapidly sequestered from brain ISF in vivo and bind GM1 ganglioside on cellular membranes. Neuron 82, 308–319. 24685176 10.1016/j.neuron.2014.02.027 PMC4129520

[92] Ehehalt R , Keller P , Haass C , Thiele C and Simons K (2003) Amyloidogenic processing of the Alzheimer beta‐amyloid precursor protein depends on lipid rafts. J Cell Biol 160, 113–123. 12515826 10.1083/jcb.200207113 PMC2172747

[93] Marquer C , Devauges V , Cossec JC , Liot G , Lecart S , Saudou F , Duyckaerts C , Leveque‐Fort S and Potier MC (2011) Local cholesterol increase triggers amyloid precursor protein‐Bace1 clustering in lipid rafts and rapid endocytosis. FASEB J 25, 1295–1305. 21257714 10.1096/fj.10-168633

[94] Xiong H , Callaghan D , Jones A , Walker DG , Lue LF , Beach TG , Sue LI , Woulfe J , Xu H , Stanimirovic DB *et al*. (2008) Cholesterol retention in Alzheimer's brain is responsible for high beta‐ and gamma‐secretase activities and Abeta production. Neurobiol Dis 29, 422–437. 18086530 10.1016/j.nbd.2007.10.005 PMC2720683

[95] Glockner F , Meske V , Lutjohann D and Ohm TG (2011) Dietary cholesterol and its effect on tau protein: a study in apolipoprotein E‐deficient and P301L human tau mice. J Neuropathol Exp Neurol 70, 292–301. 21412171 10.1097/NEN.0b013e318212f185

[96] Glockner F and Ohm TG (2014) Tau pathology induces intraneuronal cholesterol accumulation. J Neuropathol Exp Neurol 73, 846–854. 25101701 10.1097/NEN.0000000000000103

[97] Wu M , Zhai Y , Liang X , Chen W , Lin R , Ma L , Huang Y , Zhao D , Liang Y , Zhao W *et al*. (2022) Connecting the dots between hypercholesterolemia and Alzheimer's disease: a potential mechanism based on 27‐hydroxycholesterol. Front Neurosci 16, 842814. 35464321 10.3389/fnins.2022.842814 PMC9021879

[98] Poitelon Y , Kopec AM and Belin S (2020) Myelin fat facts: An overview of lipids and fatty acid metabolism. Cell 9, 812. 10.3390/cells9040812 PMC7226731 32230947

[99] Norton WT and Poduslo SE (1973) Myelination in rat brain: changes in myelin composition during brain maturation. J Neurochem 21, 759–773. 4754856 10.1111/j.1471-4159.1973.tb07520.x

[100] O'Brien JS (1965) Stability of the myelin membrane. Science 147, 1099–1107. 14242030 10.1126/science.147.3662.1099

[101] O'Brien JS and Sampson EL (1965) Lipid composition of the normal human brain: gray matter, white matter, and myelin. J Lipid Res 6, 537–544. 5865382

[102] Camargo N , Goudriaan A , van Deijk AF , Otte WM , Brouwers JF , Lodder H , Gutmann DH , Nave KA , Dijkhuizen RM , Mansvelder HD *et al*. (2017) Oligodendroglial myelination requires astrocyte‐derived lipids. PLoS Biol 15, e1002605. 28549068 10.1371/journal.pbio.1002605 PMC5446120

[103] Dietschy JM and Turley SD (2004) Thematic review series: brain lipids. Cholesterol metabolism in the central nervous system during early development and in the mature animal. J Lipid Res 45, 1375–1397. 15254070 10.1194/jlr.R400004-JLR200

[104] Koper JW , Lopes‐Cardozo M and Van Golde LM (1981) Preferential utilization of ketone bodies for the synthesis of myelin cholesterol in vivo. Biochim Biophys Acta 666, 411–417. 7326251 10.1016/0005-2760(81)90300-3

[105] Saher G , Brugger B , Lappe‐Siefke C , Mobius W , Tozawa R , Wehr MC , Wieland F , Ishibashi S and Nave KA (2005) High cholesterol level is essential for myelin membrane growth. Nat Neurosci 8, 468–475. 15793579 10.1038/nn1426

[106] Demel RA and De Kruyff B (1976) The function of sterols in membranes. Biochim Biophys Acta 457, 109–132. 184844 10.1016/0304-4157(76)90008-3

[107] Marcus J and Popko B (2002) Galactolipids are molecular determinants of myelin development and axo‐glial organization. Biochim Biophys Acta 1573, 406–413. 12417425 10.1016/s0304-4165(02)00410-5

[108] Maier O , Hoekstra D and Baron W (2008) Polarity development in oligodendrocytes: sorting and trafficking of myelin components. J Mol Neurosci 35, 35–53. 18172773 10.1007/s12031-007-9024-8

[109] Aggarwal S , Yurlova L and Simons M (2011) Central nervous system myelin: structure, synthesis and assembly. Trends Cell Biol 21, 585–593. 21763137 10.1016/j.tcb.2011.06.004

[110] Bakhti M , Aggarwal S and Simons M (2014) Myelin architecture: zippering membranes tightly together. Cell Mol Life Sci 71, 1265–1277. 24165921 10.1007/s00018-013-1492-0 PMC11113231

[111] Saadat L , Dupree JL , Kilkus J , Han X , Traka M , Proia RL , Dawson G and Popko B (2010) Absence of oligodendroglial glucosylceramide synthesis does not result in CNS myelin abnormalities or alter the dysmyelinating phenotype of CGT‐deficient mice. Glia 58, 391–398. 19705459 10.1002/glia.20930 PMC2807477

[112] Luoma AM , Kuo F , Cakici O , Crowther MN , Denninger AR , Avila RL , Brites P and Kirschner DA (2015) Plasmalogen phospholipids protect internodal myelin from oxidative damage. Free Radic Biol Med 84, 296–310. 25801291 10.1016/j.freeradbiomed.2015.03.012

[113] Chrast R , Saher G , Nave KA and Verheijen MH (2011) Lipid metabolism in myelinating glial cells: lessons from human inherited disorders and mouse models. J Lipid Res 52, 419–434. 21062955 10.1194/jlr.R009761 PMC3035679

[114] Schmitt S , Castelvetri LC and Simons M (2015) Metabolism and functions of lipids in myelin. Biochim Biophys Acta 1851, 999–1005. 25542507 10.1016/j.bbalip.2014.12.016

[115] Sassa T and Kihara A (2014) Metabolism of very long‐chain fatty acids: genes and pathophysiology. Biomol Ther 22, 83–92. 10.4062/biomolther.2014.017 PMC3975470 24753812

[116] Imgrund S , Hartmann D , Farwanah H , Eckhardt M , Sandhoff R , Degen J , Gieselmann V , Sandhoff K and Willecke K (2009) Adult ceramide synthase 2 (CERS2)‐deficient mice exhibit myelin sheath defects, cerebellar degeneration, and hepatocarcinomas. J Biol Chem 284, 33549–33560. 19801672 10.1074/jbc.M109.031971 PMC2785198

[117] Brites P , Mooyer PA , El Mrabet L , Waterham HR and Wanders RJ (2009) Plasmalogens participate in very‐long‐chain fatty acid‐induced pathology. Brain 132, 482–492. 19022859 10.1093/brain/awn295

[118] Kassmann CM , Lappe‐Siefke C , Baes M , Brugger B , Mildner A , Werner HB , Natt O , Michaelis T , Prinz M , Frahm J *et al*. (2007) Axonal loss and neuroinflammation caused by peroxisome‐deficient oligodendrocytes. Nat Genet 39, 969–976. 17643102 10.1038/ng2070

[119] Khan M , Singh J , Gilg AG , Uto T and Singh I (2010) Very long‐chain fatty acid accumulation causes lipotoxic response via 5‐lipoxygenase in cerebral adrenoleukodystrophy. J Lipid Res 51, 1685–1695. 20173212 10.1194/jlr.M002329 PMC2882744

[120] Dimas P , Montani L , Pereira JA , Moreno D , Trotzmuller M , Gerber J , Semenkovich CF , Kofeler HC and Suter U (2019) CNS myelination and remyelination depend on fatty acid synthesis by oligodendrocytes. Elife 8, e44702. 31063129 10.7554/eLife.44702 PMC6504237

[121] Saher G and Simons M (2010) Cholesterol and myelin biogenesis. Subcell Biochem 51, 489–508. 20213556 10.1007/978-90-481-8622-8_18

[122] Salles J , Sargueil F , Knoll‐Gellida A , Witters LA , Shy M , Jiang H , Cassagne C and Garbay B (2002) Fatty acid synthase expression during peripheral nervous system myelination. Brain Res Mol Brain Res 101, 52–58. 12007831 10.1016/s0169-328x(02)00161-4

[123] Verheijen MH , Camargo N , Verdier V , Nadra K , de Preux Charles AS , Medard JJ , Luoma A , Crowther M , Inouye H , Shimano H *et al*. (2009) SCAP is required for timely and proper myelin membrane synthesis. Proc Natl Acad Sci U S A 106, 21383–21388. 19948958 10.1073/pnas.0905633106 PMC2795508

[124] Hamilton JA and Brunaldi K (2007) A model for fatty acid transport into the brain. J Mol Neurosci 33, 12–17. 17901540 10.1007/s12031-007-0050-3

[125] Mitchell RW and Hatch GM (2011) Fatty acid transport into the brain: of fatty acid fables and lipid tails. Prostaglandins Leukot Essent Fatty Acids 85, 293–302. 21816594 10.1016/j.plefa.2011.04.007

[126] Zhang W , Chen R , Yang T , Xu N , Chen J , Gao Y and Stetler RA (2018) Fatty acid transporting proteins: roles in brain development, aging, and stroke. Prostaglandins Leukot Essent Fatty Acids 136, 35–45. 28457600 10.1016/j.plefa.2017.04.004 PMC5650946

[127] Sharifi K , Ebrahimi M , Kagawa Y , Islam A , Tuerxun T , Yasumoto Y , Hara T , Yamamoto Y , Miyazaki H , Tokuda N *et al*. (2013) Differential expression and regulatory roles of FABP5 and FABP7 in oligodendrocyte lineage cells. Cell Tissue Res 354, 683–695. 24114376 10.1007/s00441-013-1730-7

[128] Panov A , Orynbayeva Z , Vavilin V and Lyakhovich V (2014) Fatty acids in energy metabolism of the central nervous system. Biomed Res Int 2014, 472459. 24883315 10.1155/2014/472459 PMC4026875

[129] Schonfeld P and Reiser G (2013) Why does brain metabolism not favor burning of fatty acids to provide energy? Reflections on disadvantages of the use of free fatty acids as fuel for brain. J Cereb Blood Flow Metab 33, 1493–1499. 23921897 10.1038/jcbfm.2013.128 PMC3790936

[130] Funfschilling U , Supplie LM , Mahad D , Boretius S , Saab AS , Edgar J , Brinkmann BG , Kassmann CM , Tzvetanova ID , Mobius W *et al*. (2012) Glycolytic oligodendrocytes maintain myelin and long‐term axonal integrity. Nature 485, 517–521. 22622581 10.1038/nature11007 PMC3613737

[131] Verheijen MH , Chrast R , Burrola P and Lemke G (2003) Local regulation of fat metabolism in peripheral nerves. Genes Dev 17, 2450–2464. 14522948 10.1101/gad.1116203 PMC218081

[132] Roher AE , Weiss N , Kokjohn TA , Kuo YM , Kalback W , Anthony J , Watson D , Luehrs DC , Sue L , Walker D *et al*. (2002) Increased a beta peptides and reduced cholesterol and myelin proteins characterize white matter degeneration in Alzheimer's disease. Biochemistry 41, 11080–11090. 12220172 10.1021/bi026173d

[133] Behrendt G , Baer K , Buffo A , Curtis MA , Faull RL , Rees MI , Gotz M and Dimou L (2013) Dynamic changes in myelin aberrations and oligodendrocyte generation in chronic amyloidosis in mice and men. Glia 61, 273–286. 23090919 10.1002/glia.22432

[134] Sobue A , Komine O , Hara Y , Endo F , Mizoguchi H , Watanabe S , Murayama S , Saito T , Saido TC , Sahara N *et al*. (2021) Microglial gene signature reveals loss of homeostatic microglia associated with neurodegeneration of Alzheimer's disease. Acta Neuropathol Commun 9, 1. 33402227 10.1186/s40478-020-01099-x PMC7786928

[135] Gagyi E , Kormos B , Castellanos KJ , Valyi‐Nagy K , Korneff D , LoPresti P , Woltjer R and Valyi‐Nagy T (2012) Decreased oligodendrocyte nuclear diameter in Alzheimer's disease and Lewy body dementia. Brain Pathol 22, 803–810. 22429607 10.1111/j.1750-3639.2012.00595.x PMC4181948

[136] Mathys H , Davila‐Velderrain J , Peng Z , Gao F , Mohammadi S , Young JZ , Menon M , He L , Abdurrob F , Jiang X *et al*. (2019) Single‐cell transcriptomic analysis of Alzheimer's disease. Nature 570, 332–337. 31042697 10.1038/s41586-019-1195-2 PMC6865822

[137] Pak K , Chan SL and Mattson MP (2003) Presenilin‐1 mutation sensitizes oligodendrocytes to glutamate and amyloid toxicities, and exacerbates white matter damage and memory impairment in mice. Neuromolecular Med 3, 53–64. 12665676 10.1385/NMM:3:1:53

[138] Desai MK , Mastrangelo MA , Ryan DA , Sudol KL , Narrow WC and Bowers WJ (2010) Early oligodendrocyte/myelin pathology in Alzheimer's disease mice constitutes a novel therapeutic target. Am J Pathol 177, 1422–1435. 20696774 10.2353/ajpath.2010.100087 PMC2928974

[139] Chen JF , Liu K , Hu B , Li RR , Xin W , Chen H , Wang F , Chen L , Li RX , Ren SY *et al*. (2021) Enhancing myelin renewal reverses cognitive dysfunction in a murine model of Alzheimer's disease. Neuron 109, 2292–2307.e5. 34102111 10.1016/j.neuron.2021.05.012 PMC8298291

[140] Chen JF , Wang F , Huang NX , Xiao L and Mei F (2022) Oligodendrocytes and myelin: active players in neurodegenerative brains? Dev Neurobiol 82, 160–174. 35081276 10.1002/dneu.22867

[141] Depp C , Sun T , Sasmita AO , Spieth L , Berghoff SA , Nazarenko T , Overhoff K , Steixner‐Kumar AA , Subramanian S , Arinrad S *et al*. (2023) Myelin dysfunction drives amyloid‐beta deposition in models of Alzheimer's disease. Nature 618, 349–357. 37258678 10.1038/s41586-023-06120-6 PMC10247380

[142] Kenigsbuch M , Bost P , Halevi S , Chang Y , Chen S , Ma Q , Hajbi R , Schwikowski B , Bodenmiller B , Fu H *et al*. (2022) A shared disease‐associated oligodendrocyte signature among multiple CNS pathologies. Nat Neurosci 25, 876–886. 35760863 10.1038/s41593-022-01104-7 PMC9724210

[143] Pandey S , Shen K , Lee SH , Shen YA , Wang Y , Otero‐Garcia M , Kotova N , Vito ST , Laufer BI , Newton DF *et al*. (2022) Disease‐associated oligodendrocyte responses across neurodegenerative diseases. Cell Rep 40, 111189. 36001972 10.1016/j.celrep.2022.111189

[144] Park H , Cho B , Kim H , Saito T , Saido TC , Won KJ and Kim J (2023) Single‐cell RNA‐sequencing identifies disease‐associated oligodendrocytes in male APP NL‐G‐F and 5XFAD mice. Nat Commun 14, 802. 36781874 10.1038/s41467-023-36519-8 PMC9925742

[145] Shi F , Liu B , Zhou Y , Yu C and Jiang T (2009) Hippocampal volume and asymmetry in mild cognitive impairment and Alzheimer's disease: meta‐analyses of MRI studies. Hippocampus 19, 1055–1064. 19309039 10.1002/hipo.20573

[146] Barnes J , Bartlett JW , van de Pol LA , Loy CT , Scahill RI , Frost C , Thompson P and Fox NC (2009) A meta‐analysis of hippocampal atrophy rates in Alzheimer's disease. Neurobiol Aging 30, 1711–1723. 18346820 10.1016/j.neurobiolaging.2008.01.010 PMC2773132

[147] Fox NC , Warrington EK , Freeborough PA , Hartikainen P , Kennedy AM , Stevens JM and Rossor MN (1996) Presymptomatic hippocampal atrophy in Alzheimer's disease. A longitudinal MRI study. Brain 119 (Pt 6), 2001–2007. 9010004 10.1093/brain/119.6.2001

[148] Jack CR Jr , Petersen RC , Xu Y , O'Brien PC , Smith GE , Ivnik RJ , Boeve BF , Tangalos EG and Kokmen E (2000) Rates of hippocampal atrophy correlate with change in clinical status in aging and AD. Neurology 55, 484–489. 10953178 10.1212/wnl.55.4.484 PMC2724764

[149] McDonald CR , Gharapetian L , McEvoy LK , Fennema‐Notestine C , Hagler DJ Jr , Holland D , Dale AM and Alzheimer's Disease Neuroimaging Initiative (2012) Relationship between regional atrophy rates and cognitive decline in mild cognitive impairment. Neurobiol Aging 33, 242–253. 20471718 10.1016/j.neurobiolaging.2010.03.015 PMC2923665

[150] Bastos‐Leite AJ , van der Flier WM , van Straaten EC , Staekenborg SS , Scheltens P and Barkhof F (2007) The contribution of medial temporal lobe atrophy and vascular pathology to cognitive impairment in vascular dementia. Stroke 38, 3182–3185. 17962598 10.1161/STROKEAHA.107.490102

[151] Laakso MP , Partanen K , Riekkinen P , Lehtovirta M , Helkala EL , Hallikainen M , Hanninen T , Vainio P and Soininen H (1996) Hippocampal volumes in Alzheimer's disease, Parkinson's disease with and without dementia, and in vascular dementia: an MRI study. Neurology 46, 678–681. 8618666 10.1212/wnl.46.3.678

[152] Chan D , Fox NC , Scahill RI , Crum WR , Whitwell JL , Leschziner G , Rossor AM , Stevens JM , Cipolotti L and Rossor MN (2001) Patterns of temporal lobe atrophy in semantic dementia and Alzheimer's disease. Ann Neurol 49, 433–442. 11310620

[153] Pini L , Pievani M , Bocchetta M , Altomare D , Bosco P , Cavedo E , Galluzzi S , Marizzoni M and Frisoni GB (2016) Brain atrophy in Alzheimer's disease and aging. Ageing Res Rev 30, 25–48. 26827786 10.1016/j.arr.2016.01.002

[154] van de Pol LA , Hensel A , van der Flier WM , Visser PJ , Pijnenburg YA , Barkhof F , Gertz HJ and Scheltens P (2006) Hippocampal atrophy on MRI in frontotemporal lobar degeneration and Alzheimer's disease. J Neurol Neurosurg Psychiatry 77, 439–442. 16306153 10.1136/jnnp.2005.075341 PMC2077497

[155] Bobinski M , de Leon MJ , Wegiel J , Desanti S , Convit A , Saint Louis LA , Rusinek H and Wisniewski HM (2000) The histological validation of post mortem magnetic resonance imaging‐determined hippocampal volume in Alzheimer's disease. Neuroscience 95, 721–725. 10670438 10.1016/s0306-4522(99)00476-5

[156] Gosche KM , Mortimer JA , Smith CD , Markesbery WR and Snowdon DA (2002) Hippocampal volume as an index of Alzheimer neuropathology: findings from the nun study. Neurology 58, 1476–1482. 12034782 10.1212/wnl.58.10.1476

[157] Jack CR Jr , Dickson DW , Parisi JE , Xu YC , Cha RH , O'Brien PC , Edland SD , Smith GE , Boeve BF , Tangalos EG *et al*. (2002) Antemortem MRI findings correlate with hippocampal neuropathology in typical aging and dementia. Neurology 58, 750–757. 11889239 10.1212/wnl.58.5.750 PMC2745935

[158] Brown WR and Thore CR (2011) Review: cerebral microvascular pathology in ageing and neurodegeneration. Neuropathol Appl Neurobiol 37, 56–74. 20946471 10.1111/j.1365-2990.2010.01139.x PMC3020267

[159] Schuff N , Matsumoto S , Kmiecik J , Studholme C , Du A , Ezekiel F , Miller BL , Kramer JH , Jagust WJ , Chui HC *et al*. (2009) Cerebral blood flow in ischemic vascular dementia and Alzheimer's disease, measured by arterial spin‐labeling magnetic resonance imaging. Alzheimers Dement 5, 454–462. 19896584 10.1016/j.jalz.2009.04.1233 PMC2802181

[160] Brickman AM (2013) Contemplating Alzheimer's disease and the contribution of white matter hyperintensities. Curr Neurol Neurosci Rep 13, 415. 24190781 10.1007/s11910-013-0415-7 PMC3874404

[161] Brickman AM , Provenzano FA , Muraskin J , Manly JJ , Blum S , Apa Z , Stern Y , Brown TR , Luchsinger JA and Mayeux R (2012) Regional white matter hyperintensity volume, not hippocampal atrophy, predicts incident Alzheimer disease in the community. Arch Neurol 69, 1621–1627. 22945686 10.1001/archneurol.2012.1527 PMC3597387

[162] Brickman AM , Zahodne LB , Guzman VA , Narkhede A , Meier IB , Griffith EY , Provenzano FA , Schupf N , Manly JJ , Stern Y *et al*. (2015) Reconsidering harbingers of dementia: progression of parietal lobe white matter hyperintensities predicts Alzheimer's disease incidence. Neurobiol Aging 36, 27–32. 25155654 10.1016/j.neurobiolaging.2014.07.019 PMC4268124

[163] Tosto G , Zimmerman ME , Carmichael OT , Brickman AM and Alzheimer's Disease Neuroimaging Initiative (2014) Predicting aggressive decline in mild cognitive impairment: the importance of white matter hyperintensities. JAMA Neurol 71, 872–877. 24821476 10.1001/jamaneurol.2014.667 PMC4107926

[164] Brickman AM , Schupf N , Manly JJ , Stern Y , Luchsinger JA , Provenzano FA , Narkhede A , Razlighi Q , Collins‐Praino L , Artero S *et al*. (2014) APOE epsilon4 and risk for Alzheimer's disease: do regionally distributed white matter hyperintensities play a role? Alzheimers Dement 10, 619–629. 25304991 10.1016/j.jalz.2014.07.155 PMC4252241

[165] Lee S , Viqar F , Zimmerman ME , Narkhede A , Tosto G , Benzinger TL , Marcus DS , Fagan AM , Goate A , Fox NC *et al*. (2016) White matter hyperintensities are a core feature of Alzheimer's disease: evidence from the dominantly inherited Alzheimer network. Ann Neurol 79, 929–939. 27016429 10.1002/ana.24647 PMC4884146

[166] Scott JA , Braskie MN , Tosun D , Thompson PM , Weiner M , DeCarli C , Carmichael OT and Alzheimer's Disease Neuroimaging Initiative (2015) Cerebral amyloid and hypertension are independently associated with white matter lesions in elderly. Front Aging Neurosci 7, 221. 26648866 10.3389/fnagi.2015.00221 PMC4664630

[167] Tosto G , Zimmerman ME , Hamilton JL , Carmichael OT , Brickman AM and Alzheimer's Disease Neuroimaging Initiative (2015) The effect of white matter hyperintensities on neurodegeneration in mild cognitive impairment. Alzheimers Dement 11, 1510–1519. 26079417 10.1016/j.jalz.2015.05.014 PMC4677059

[168] Nasrabady SE , Rizvi B , Goldman JE and Brickman AM (2018) White matter changes in Alzheimer's disease: a focus on myelin and oligodendrocytes. Acta Neuropathol Commun 6, 22. 29499767 10.1186/s40478-018-0515-3 PMC5834839

[169] Zhao Z , Nelson AR , Betsholtz C and Zlokovic BV (2015) Establishment and dysfunction of the blood‐brain barrier. Cell 163, 1064–1078. 26590417 10.1016/j.cell.2015.10.067 PMC4655822

[170] Fischer FU , Wolf D , Scheurich A , Fellgiebel A and Alzheimer's Disease Neuroimaging Initiative (2015) Altered whole‐brain white matter networks in preclinical Alzheimer's disease. Neuroimage Clin 8, 660–666. 26288751 10.1016/j.nicl.2015.06.007 PMC4536470

[171] Desai MK , Sudol KL , Janelsins MC , Mastrangelo MA , Frazer ME and Bowers WJ (2009) Triple‐transgenic Alzheimer's disease mice exhibit region‐specific abnormalities in brain myelination patterns prior to appearance of amyloid and tau pathology. Glia 57, 54–65. 18661556 10.1002/glia.20734 PMC2584762

[172] McAleese KE , Miah M , Graham S , Hadfield GM , Walker L , Johnson M , Colloby SJ , Thomas AJ , DeCarli C , Koss D *et al*. (2021) Frontal white matter lesions in Alzheimer's disease are associated with both small vessel disease and AD‐associated cortical pathology. Acta Neuropathol 142, 937–950. 34608542 10.1007/s00401-021-02376-2 PMC8568857

[173] McAleese KE , Walker L , Graham S , Moya ELJ , Johnson M , Erskine D , Colloby SJ , Dey M , Martin‐Ruiz C , Taylor JP *et al*. (2017) Parietal white matter lesions in Alzheimer's disease are associated with cortical neurodegenerative pathology, but not with small vessel disease. Acta Neuropathol 134, 459–473. 28638989 10.1007/s00401-017-1738-2 PMC5563333

[174] Erten‐Lyons D , Woltjer R , Kaye J , Mattek N , Dodge HH , Green S , Tran H , Howieson DB , Wild K and Silbert LC (2013) Neuropathologic basis of white matter hyperintensity accumulation with advanced age. Neurology 81, 977–983. 23935177 10.1212/WNL.0b013e3182a43e45 PMC3888199

[175] Gouw AA , Seewann A , Vrenken H , van der Flier WM , Rozemuller JM , Barkhof F , Scheltens P and Geurts JJ (2008) Heterogeneity of white matter hyperintensities in Alzheimer's disease: post‐mortem quantitative MRI and neuropathology. Brain 131, 3286–3298. 18927145 10.1093/brain/awn265

[176] Scheltens P , Barkhof F , Leys D , Wolters EC , Ravid R and Kamphorst W (1995) Histopathologic correlates of white matter changes on MRI in Alzheimer's disease and normal aging. Neurology 45, 883–888. 7746401 10.1212/wnl.45.5.883

[177] Braak H and Braak E (1996) Development of Alzheimer‐related neurofibrillary changes in the neocortex inversely recapitulates cortical myelogenesis. Acta Neuropathol 92, 197–201. 8841666 10.1007/s004010050508

[178] Bartzokis G , Cummings JL , Sultzer D , Henderson VW , Nuechterlein KH and Mintz J (2003) White matter structural integrity in healthy aging adults and patients with Alzheimer disease: a magnetic resonance imaging study. Arch Neurol 60, 393–398. 12633151 10.1001/archneur.60.3.393

[179] Benitez A , Fieremans E , Jensen JH , Falangola MF , Tabesh A , Ferris SH and Helpern JA (2014) White matter tract integrity metrics reflect the vulnerability of late‐myelinating tracts in Alzheimer's disease. Neuroimage Clin 4, 64–71. 24319654 10.1016/j.nicl.2013.11.001 PMC3853114

[180] Gao J , Cheung RT , Lee TM , Chu LW , Chan YS , Mak HK , Zhang JX , Qiu D , Fung G and Cheung C (2011) Possible retrogenesis observed with fiber tracking: an anteroposterior pattern of white matter disintegrity in normal aging and Alzheimer's disease. J Alzheimers Dis 26, 47–58. 21558648 10.3233/JAD-2011-101788

[181] Reisberg B , Franssen EH , Hasan SM , Monteiro I , Boksay I , Souren LE , Kenowsky S , Auer SR , Elahi S and Kluger A (1999) Retrogenesis: clinical, physiologic, and pathologic mechanisms in brain aging, Alzheimer's and other dementing processes. Eur Arch Psychiatry Clin Neurosci 249 (Suppl 3), 28–36. 10.1007/pl00014170 10654097

[182] Fitzpatrick AL , Kuller LH , Lopez OL , Diehr P , O'Meara ES , Longstreth WT Jr and Luchsinger JA (2009) Midlife and late‐life obesity and the risk of dementia: cardiovascular health study. Arch Neurol 66, 336–342. 19273752 10.1001/archneurol.2008.582 PMC3513375

[183] Gustafson D , Rothenberg E , Blennow K , Steen B and Skoog I (2003) An 18‐year follow‐up of overweight and risk of Alzheimer disease. Arch Intern Med 163, 1524–1528. 12860573 10.1001/archinte.163.13.1524

[184] Hayden KM , Zandi PP , Lyketsos CG , Khachaturian AS , Bastian LA , Charoonruk G , Tschanz JT , Norton MC , Pieper CF , Munger RG *et al*. (2006) Vascular risk factors for incident Alzheimer disease and vascular dementia: the Cache County study. Alzheimer Dis Assoc Disord 20, 93–100. 16772744 10.1097/01.wad.0000213814.43047.86

[185] Kivipelto M , Ngandu T , Fratiglioni L , Viitanen M , Kareholt I , Winblad B , Helkala EL , Tuomilehto J , Soininen H and Nissinen A (2005) Obesity and vascular risk factors at midlife and the risk of dementia and Alzheimer disease. Arch Neurol 62, 1556–1560. 16216938 10.1001/archneur.62.10.1556

[186] Ma Y , Ajnakina O , Steptoe A and Cadar D (2020) Higher risk of dementia in English older individuals who are overweight or obese. Int J Epidemiol 49, 1353–1365. 32575116 10.1093/ije/dyaa099 PMC7660153

[187] Nam KN , Mounier A , Wolfe CM , Fitz NF , Carter AY , Castranio EL , Kamboh HI , Reeves VL , Wang J , Han X *et al*. (2017) Effect of high fat diet on phenotype, brain transcriptome and lipidome in Alzheimer's model mice. Sci Rep 7, 4307. 28655926 10.1038/s41598-017-04412-2 PMC5487356

[188] Anstey KJ , Cherbuin N , Budge M and Young J (2011) Body mass index in midlife and late‐life as a risk factor for dementia: a meta‐analysis of prospective studies. Obes Rev 12, e426–e437. 21348917 10.1111/j.1467-789X.2010.00825.x

[189] de Bem AF , Krolow R , Farias HR , de Rezende VL , Gelain DP , Moreira JCF , Duarte J and de Oliveira J (2020) Animal models of metabolic disorders in the study of neurodegenerative diseases: an overview. Front Neurosci 14, 604150. 33536868 10.3389/fnins.2020.604150 PMC7848140

[190] Busquets O , Ettcheto M , Pallas M , Beas‐Zarate C , Verdaguer E , Auladell C , Folch J and Camins A (2017) Long‐term exposition to a high fat diet favors the appearance of beta‐amyloid depositions in the brain of C57BL/6J mice. A potential model of sporadic Alzheimer's disease. Mech Ageing Dev 162, 38–45. 27863851 10.1016/j.mad.2016.11.002

[191] Nakandakari S , Munoz VR , Kuga GK , Gaspar RC , Sant'Ana MR , Pavan ICB , da Silva LGS , Morelli AP , Simabuco FM , da Silva ASR *et al*. (2019) Short‐term high‐fat diet modulates several inflammatory, ER stress, and apoptosis markers in the hippocampus of young mice. Brain Behav Immun 79, 284–293. 30797044 10.1016/j.bbi.2019.02.016

[192] Bittencourt A , Brum PO , Ribeiro CT , Gasparotto J , Bortolin RC , de Vargas AR , Heimfarth L , de Almeida RF , Moreira JCF , de Oliveira J *et al*. (2022) High fat diet‐induced obesity causes a reduction in brain tyrosine hydroxylase levels and non‐motor features in rats through metabolic dysfunction, neuroinflammation and oxidative stress. Nutr Neurosci 25, 1026–1040. 33078695 10.1080/1028415X.2020.1831261

[193] Denver P , Gault VA and McClean PL (2018) Sustained high‐fat diet modulates inflammation, insulin signalling and cognition in mice and a modified xenin peptide ameliorates neuropathology in a chronic high‐fat model. Diabetes Obes Metab 20, 1166–1175. 29316242 10.1111/dom.13210

[194] Salameh TS , Mortell WG , Logsdon AF , Butterfield DA and Banks WA (2019) Disruption of the hippocampal and hypothalamic blood‐brain barrier in a diet‐induced obese model of type II diabetes: prevention and treatment by the mitochondrial carbonic anhydrase inhibitor, topiramate. Fluids Barriers CNS 16, 1. 30616618 10.1186/s12987-018-0121-6 PMC6323732

[195] Zhan R , Zhao M , Zhou T , Chen Y , Yu W , Zhao L , Zhang T , Wang H , Yang H , Jin Y *et al*. (2018) Dapsone protects brain microvascular integrity from high‐fat diet induced LDL oxidation. Cell Death Dis 9, 683. 29880899 10.1038/s41419-018-0739-y PMC5992187

[196] Arnold SE , Lucki I , Brookshire BR , Carlson GC , Browne CA , Kazi H , Bang S , Choi BR , Chen Y , McMullen MF *et al*. (2014) High fat diet produces brain insulin resistance, synaptodendritic abnormalities and altered behavior in mice. Neurobiol Dis 67, 79–87. 24686304 10.1016/j.nbd.2014.03.011 PMC4083060

[197] Underwood EL and Thompson LT (2016) A high‐fat diet causes impairment in hippocampal memory and sex‐dependent alterations in peripheral metabolism. Neural Plast 2016, 7385314. 26819773 10.1155/2016/7385314 PMC4706969

[198] Spencer SJ , D'Angelo H , Soch A , Watkins LR , Maier SF and Barrientos RM (2017) High‐fat diet and aging interact to produce neuroinflammation and impair hippocampal‐ and amygdalar‐dependent memory. Neurobiol Aging 58, 88–101. 28719855 10.1016/j.neurobiolaging.2017.06.014 PMC5581696

[199] McLean FH , Grant C , Morris AC , Horgan GW , Polanski AJ , Allan K , Campbell FM , Langston RF and Williams LM (2018) Rapid and reversible impairment of episodic memory by a high‐fat diet in mice. Sci Rep 8, 11976. 30097632 10.1038/s41598-018-30265-4 PMC6086894

[200] Lizarbe B , Soares AF , Larsson S and Duarte JMN (2018) Neurochemical modifications in the hippocampus, cortex and hypothalamus of mice exposed to long‐term high‐fat diet. Front Neurosci 12, 985. 30670942 10.3389/fnins.2018.00985 PMC6331468

[201] Morganstern I , Ye Z , Liang S , Fagan S and Leibowitz SF (2012) Involvement of cholinergic mechanisms in the behavioral effects of dietary fat consumption. Brain Res 1470, 24–34. 22765913 10.1016/j.brainres.2012.06.004 PMC3418966

[202] Takeda S , Sato N , Uchio‐Yamada K , Sawada K , Kunieda T , Takeuchi D , Kurinami H , Shinohara M , Rakugi H and Morishita R (2010) Diabetes‐accelerated memory dysfunction via cerebrovascular inflammation and Abeta deposition in an Alzheimer mouse model with diabetes. Proc Natl Acad Sci U S A 107, 7036–7041. 20231468 10.1073/pnas.1000645107 PMC2872449

[203] Shinohara M , Kikuchi M , Onishi‐Takeya M , Tashiro Y , Suzuki K , Noda Y , Takeda S , Mukouzono M , Nagano S , Fukumori A *et al*. (2021) Upregulated expression of a subset of genes in APP;Ob/Ob mice: evidence of an interaction between diabetes‐linked obesity and Alzheimer's disease. FASEB Bioadv 3, 323–333. 33977233 10.1096/fba.2020-00151 PMC8103720

[204] Nota MHC , Vreeken D , Wiesmann M , Aarts EO , Hazebroek EJ and Kiliaan AJ (2020) Obesity affects brain structure and function‐rescue by bariatric surgery? Neurosci Biobehav Rev 108, 646–657. 31794778 10.1016/j.neubiorev.2019.11.025

[205] Alarcon G , Ray S and Nagel BJ (2016) Lower working memory performance in overweight and obese adolescents is mediated by white matter microstructure. J Int Neuropsychol Soc 22, 281–292. 26708324 10.1017/S1355617715001265 PMC5642274

[206] Bolzenius JD , Laidlaw DH , Cabeen RP , Conturo TE , McMichael AR , Lane EM , Heaps JM , Salminen LE , Baker LM , Scott SE *et al*. (2015) Brain structure and cognitive correlates of body mass index in healthy older adults. Behav Brain Res 278, 342–347. 25448431 10.1016/j.bbr.2014.10.010 PMC4382378

[207] Kullmann S , Callaghan MF , Heni M , Weiskopf N , Scheffler K , Haring HU , Fritsche A , Veit R and Preissl H (2016) Specific white matter tissue microstructure changes associated with obesity. Neuroimage 125, 36–44. 26458514 10.1016/j.neuroimage.2015.10.006 PMC4692452

[208] van Bloemendaal L , Ijzerman RG , Ten Kulve JS , Barkhof F , Diamant M , Veltman DJ and van Duinkerken E (2016) Alterations in white matter volume and integrity in obesity and type 2 diabetes. Metab Brain Dis 31, 621–629. 26815786 10.1007/s11011-016-9792-3 PMC4863900

[209] Verstynen TD , Weinstein A , Erickson KI , Sheu LK , Marsland AL and Gianaros PJ (2013) Competing physiological pathways link individual differences in weight and abdominal adiposity to white matter microstructure. Neuroimage 79, 129–137. 23639257 10.1016/j.neuroimage.2013.04.075 PMC3752776

[210] Xu J , Li Y , Lin H , Sinha R and Potenza MN (2013) Body mass index correlates negatively with white matter integrity in the fornix and corpus callosum: a diffusion tensor imaging study. Hum Brain Mapp 34, 1044–1052. 22139809 10.1002/hbm.21491 PMC3314715

[211] Ronan L , Alexander‐Bloch AF , Wagstyl K , Farooqi S , Brayne C , Tyler LK , Cam CAN and Fletcher PC (2016) Obesity associated with increased brain age from midlife. Neurobiol Aging 47, 63–70. 27562529 10.1016/j.neurobiolaging.2016.07.010 PMC5082766

[212] Haltia LT , Viljanen A , Parkkola R , Kemppainen N , Rinne JO , Nuutila P and Kaasinen V (2007) Brain white matter expansion in human obesity and the recovering effect of dieting. J Clin Endocrinol Metab 92, 3278–3284. 17536002 10.1210/jc.2006-2495

[213] Hamer M and Batty GD (2019) Association of body mass index and waist‐to‐hip ratio with brain structure: UK Biobank study. Neurology 92, e594–e600. 30626649 10.1212/WNL.0000000000006879 PMC8093082

[214] Koivukangas J , Bjornholm L , Tervonen O , Miettunen J , Nordstrom T , Kiviniemi V , Maki P , Mukkala S , Moilanen I , Barnett JH *et al*. (2016) Body mass index and brain white matter structure in young adults at risk for psychosis—the Oulu Brain and Mind Study. Psychiatry Res Neuroimaging 254, 169–176. 27474847 10.1016/j.pscychresns.2016.06.016

[215] Burns JM , Johnson DK , Watts A , Swerdlow RH and Brooks WM (2010) Reduced lean mass in early Alzheimer disease and its association with brain atrophy. Arch Neurol 67, 428–433. 20385908 10.1001/archneurol.2010.38 PMC2855150

[216] Morris MC , Evans DA , Bienias JL , Tangney CC , Bennett DA , Wilson RS , Aggarwal N and Schneider J (2003) Consumption of fish and n‐3 fatty acids and risk of incident Alzheimer disease. Arch Neurol 60, 940–946. 12873849 10.1001/archneur.60.7.940

[217] Zhang Y , Chen J , Qiu J , Li Y , Wang J and Jiao J (2016) Intakes of fish and polyunsaturated fatty acids and mild‐to‐severe cognitive impairment risks: a dose‐response meta‐analysis of 21 cohort studies. Am J Clin Nutr 103, 330–340. 26718417 10.3945/ajcn.115.124081

[218] Hopperton KE , Trepanier MO , Giuliano V and Bazinet RP (2016) Brain omega‐3 polyunsaturated fatty acids modulate microglia cell number and morphology in response to intracerebroventricular amyloid‐beta 1‐40 in mice. J Neuroinflammation 13, 257. 27688126 10.1186/s12974-016-0721-5 PMC5041295

[219] Casali BT , Corona AW , Mariani MM , Karlo JC , Ghosal K and Landreth GE (2015) Omega‐3 fatty acids augment the actions of nuclear receptor agonists in a mouse model of Alzheimer's disease. J Neurosci 35, 9173–9181. 26085639 10.1523/JNEUROSCI.1000-15.2015 PMC4469742

[220] de Urquiza AM , Liu S , Sjoberg M , Zetterstrom RH , Griffiths W , Sjovall J and Perlmann T (2000) Docosahexaenoic acid, a ligand for the retinoid X receptor in mouse brain. Science 290, 2140–2144. 11118147 10.1126/science.290.5499.2140

[221] Forman BM , Chen J and Evans RM (1997) Hypolipidemic drugs, polyunsaturated fatty acids, and eicosanoids are ligands for peroxisome proliferator‐activated receptors alpha and delta. Proc Natl Acad Sci U S A 94, 4312–4317. 9113986 10.1073/pnas.94.9.4312 PMC20719

[222] Grimm MO , Mett J , Stahlmann CP , Haupenthal VJ , Blumel T , Stotzel H , Grimm HS and Hartmann T (2016) Eicosapentaenoic acid and docosahexaenoic acid increase the degradation of amyloid‐beta by affecting insulin‐degrading enzyme. Biochem Cell Biol 94, 534–542. 27813426 10.1139/bcb-2015-0149

[223] Grimm MO , Kuchenbecker J , Grosgen S , Burg VK , Hundsdorfer B , Rothhaar TL , Friess P , de Wilde MC , Broersen LM , Penke B *et al*. (2011) Docosahexaenoic acid reduces amyloid beta production via multiple pleiotropic mechanisms. J Biol Chem 286, 14028–14039. 21324907 10.1074/jbc.M110.182329 PMC3077603

[224] Quinn JF , Raman R , Thomas RG , Yurko‐Mauro K , Nelson EB , Van Dyck C , Galvin JE , Emond J , Jack CR Jr , Weiner M *et al*. (2010) Docosahexaenoic acid supplementation and cognitive decline in Alzheimer disease: a randomized trial. JAMA 304, 1903–1911. 21045096 10.1001/jama.2010.1510 PMC3259852

[225] Newman JC , Covarrubias AJ , Zhao M , Yu X , Gut P , Ng CP , Huang Y , Haldar S and Verdin E (2017) Ketogenic diet reduces midlife mortality and improves memory in aging mice. Cell Metab 26, 547–557.e8. 28877458 10.1016/j.cmet.2017.08.004 PMC5605815

[226] Van der Auwera I , Wera S , Van Leuven F and Henderson ST (2005) A ketogenic diet reduces amyloid beta 40 and 42 in a mouse model of Alzheimer's disease. Nutr Metab 2, 28. 10.1186/1743-7075-2-28 PMC1282589 16229744

[227] Kimoto A , Ohnuma T , Toda A , Takebayashi Y , Higashiyama R , Tagata Y , Ito M , Ota T , Shibata N and Arai H (2017) Medium‐chain triglycerides given in the early stage of mild‐to‐moderate Alzheimer's disease enhance memory function. Psychogeriatrics 17, 520–521. 28417540 10.1111/psyg.12257

[228] Neth BJ , Mintz A , Whitlow C , Jung Y , Solingapuram Sai K , Register TC , Kellar D , Lockhart SN , Hoscheidt S , Maldjian J *et al*. (2020) Modified ketogenic diet is associated with improved cerebrospinal fluid biomarker profile, cerebral perfusion, and cerebral ketone body uptake in older adults at risk for Alzheimer's disease: a pilot study. Neurobiol Aging 86, 54–63. 31757576 10.1016/j.neurobiolaging.2019.09.015 PMC7266642

[229] Ota M , Matsuo J , Ishida I , Takano H , Yokoi Y , Hori H , Yoshida S , Ashida K , Nakamura K , Takahashi T *et al*. (2019) Effects of a medium‐chain triglyceride‐based ketogenic formula on cognitive function in patients with mild‐to‐moderate Alzheimer's disease. Neurosci Lett 690, 232–236. 30367958 10.1016/j.neulet.2018.10.048

[230] Taylor MK , Sullivan DK , Mahnken JD , Burns JM and Swerdlow RH (2018) Feasibility and efficacy data from a ketogenic diet intervention in Alzheimer's disease. Alzheimers Dement 4, 28–36. 10.1016/j.trci.2017.11.002 PMC6021549 29955649

[231] Trapp BD and Bernsohn J (1978) Essential fatty acid deficiency and CNS myelin. Biochemical and morphological observations. J Neurol Sci 37, 249–266. 681979 10.1016/0022-510x(78)90207-1

[232] Stumpf SK , Berghoff SA , Trevisiol A , Spieth L , Duking T , Schneider LV , Schlaphoff L , Dreha‐Kulaczewski S , Bley A , Burfeind D *et al*. (2019) Ketogenic diet ameliorates axonal defects and promotes myelination in Pelizaeus‐Merzbacher disease. Acta Neuropathol 138, 147–161. 30919030 10.1007/s00401-019-01985-2 PMC6570703

